# Supplementary material for: Structure of the p53 degradation complex from HPV16
Source: Nat Commun. 2024 Feb 28;15:1842. doi: 10.1038/s41467-024-45920-w (PMC10902388; doi:10.1038/s41467-024-45920-w)
Supplement: Supplementary file 1 — Supplementary Information [file 41467_2024_45920_MOESM1_ESM.pdf]

Supplementary Information Containing

Supplementary Figures 1-33 and Supplementary Tables 1-3

Structure of the p53 degradation complex from HPV16

John C.K. Wang[&], Hannah T. Baddock[&], Amirhossein Mafi, Ian T. Foe, Matthew Bratkowski, Ting-Yu Lin, Zena D. Jensvold, Magdalena Preciado López, David Stokoe, Dan Eaton, Qi Hao[*] and Aaron H. Nile[*]

Calico Life Sciences LLC, 1170 Veterans Blvd
South San Francisco, California 94080 USA

& -- These authors contributed equally
* -- These authors jointly supervised this work

Emails: qhao@calicolabs.com and aaronnile@calicolabs.com

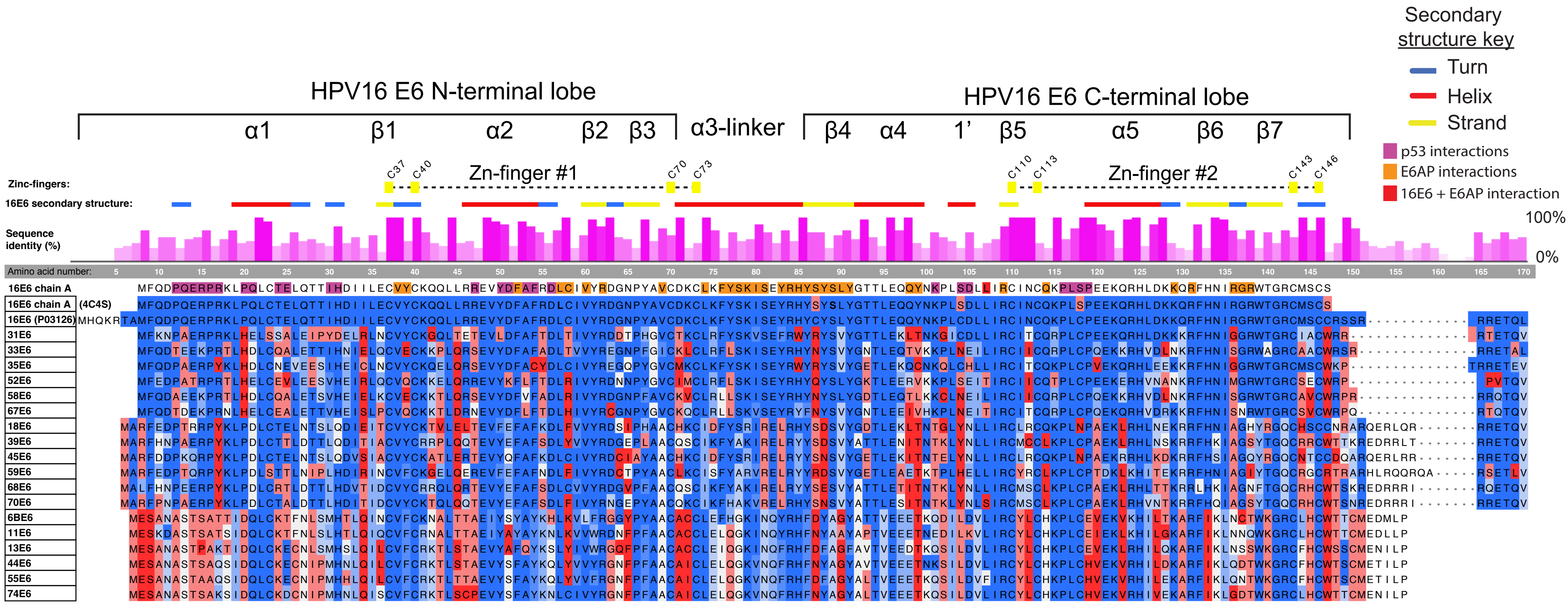

Supplementary Figure 1. HPV16 E6 interaction sites with p53 core domain and full length E6AP. Sequence alignment of HPV E6 proteins, with the cryoEM structure and Martinez-Zapien et al. *Nature* (2016) as a guide. HPV16 E6 residue similarity is color-coded (blue, similar; white, medium similarity; red, dissimilar). Interactions observed in the cryoEM structure are highlighted on the top 16E6 sequence for p53 (magenta), E6AP (orange), or both (red) above the alignment as a colored bar. The 16E6 chain A sequence represents the resolved regions of the CryoEM structure. Of note, are the Cys to Ser point mutations (4C4S, pink). The Uniprot accession numbers for the HPV E6 proteins are provided. 16E6, P03126; 31E6, P17386; 33E6, P06427; 35E6, P27228; 52E6, P36814; 58E6, P26555; 67E6, F8S5Y6; 18E6, P06463; 39E6, P24835; 45E6, P21735; 59E6, Q81964; 68E6, P54667; 70E6, P50804; 6BE6, P06462; 11E6, P04019; 13E6, Q02269; 44E6, P19710; 55E6,Q80934; 74E6, A0A7G2A3T2.

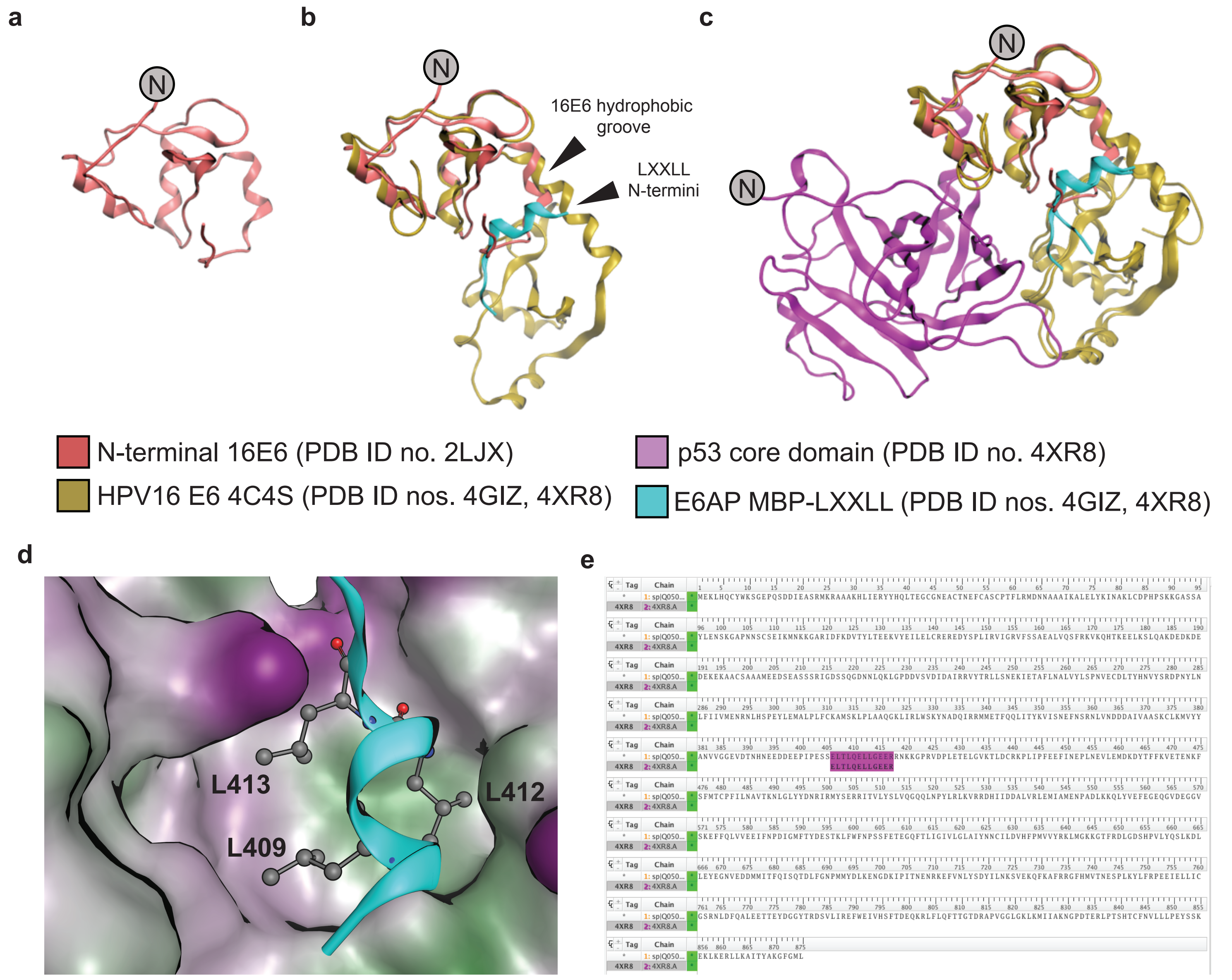

Supplementary Figure 2. Published structures of 16E6 in complex with E6AP and p53. (**a**) N-terminal Zn-finger containing lobe solved by NMR (orange-red, ribbon representation; PDB ID no. 2LJX). (**b**) Crystal structure of HPV16 E6 4C4S (gold, ribbon representation) in complex with an MBP fusion of the E6AP LXXLL peptide (blue, ribbon representation; PDB ID no. 4GIZ) superimposed onto (a). (**c**) Crystal structure of HPV 16E6 4C4S (gold, ribbon representation) bound to the MBP-LXXLL peptide (blue, ribbon representation) and p53 core domain (purple, ribbon representation; PDB ID no. 4XR8) superimposed onto (b). (**d**) Zoomed-in view of (b) visualizing the hydrophobic groove of 16E6 (green, hydrophobic; maroon, hydrophilic) bound to the MBP-LXXLL E6AP peptide (blue, ribbon representation), highlighting interacting leucine residues (gray, stick representation). (**e**) Sequence alignment of full-length human E6AP (Uniprot ID no. Q05086) and the MBP-LXXLL peptide from PDB ID no. 4XR8, highlighting the 1.4% sequence coverage (12 E6AP residues divided by 875 E6AP amino acids, purple highlight). Structures and alignment were generated using MOE. The MBP portion of the MBP-LXXLL fusion protein was hidden for clarity.

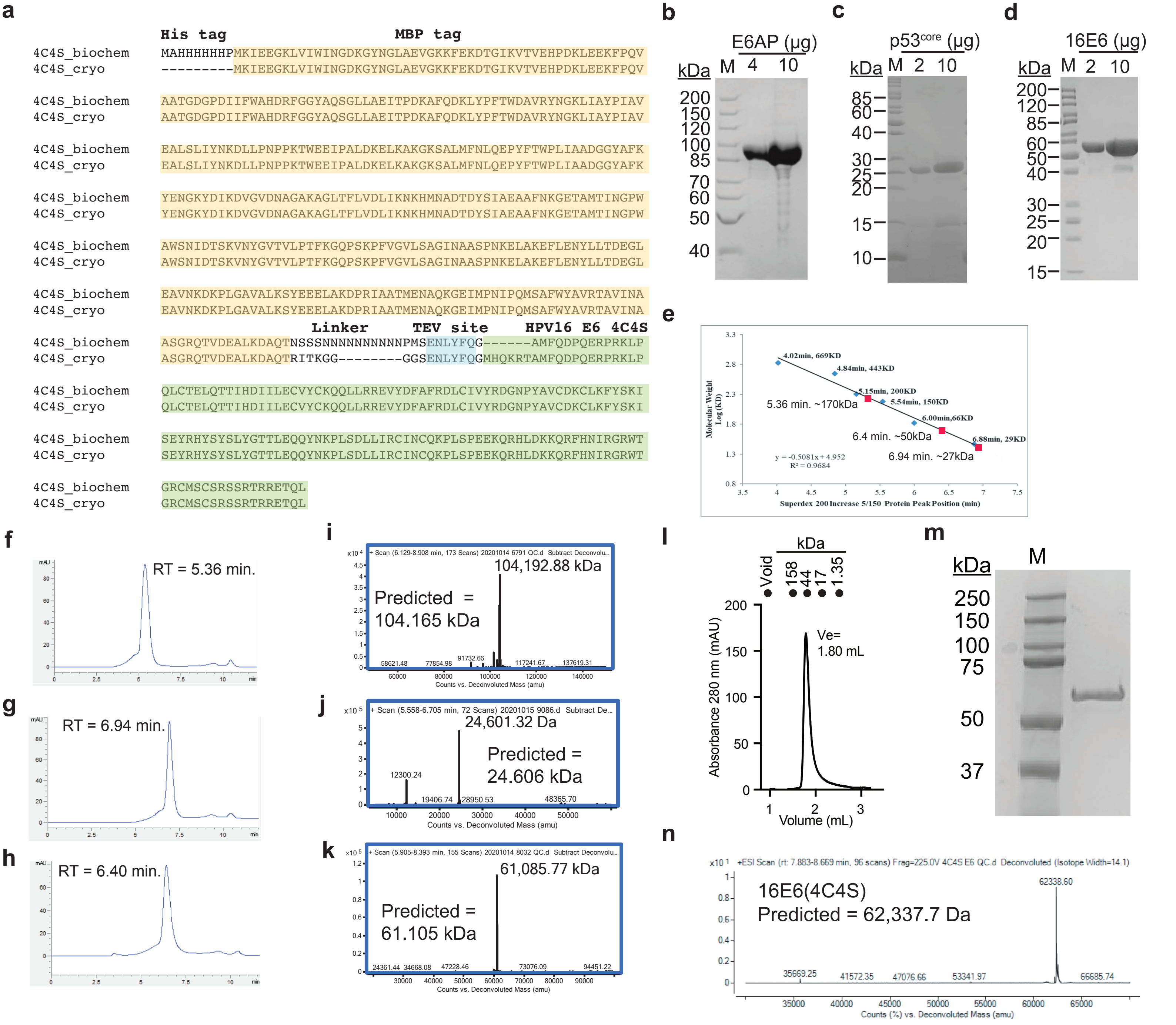

Supplementary Figure 3. Quality control of HPV16 E6 (16E6), p53^{core}, and E6AP proteins. (**a**) Sequence alignment of the primary amino acid sequence of 16E6 used for biochemical experiments (4C4S_biochem) or CryoEM (4C4S_cryo). (**b**) Resolution of E6AP (lot no. 20201012-BP6791), (**c**) p53^{core} (lot no. 20201013-BP9086), and (**d**) MBP-16E6 (lot no. 20201013-BP8032) by SDS-PAGE under reducing conditions followed by Coomassie Blue staining. Molecular weight (MW) markers, "M", are shown with the mass of loaded protein listed above each lane. (**e**) Associated MW standards (blue diamonds) resolved by analytical size exclusion chromatography (SEC) and the corresponding chromatography profile of purified (**f**) E6AP, (**g**) p53^{core}, and (**h**) MBP-16E6. Samples were resolved on a Superdex 200 Increase 5/150 GL column in 50 mM Tris-HCl (pH 7.5) and 300 mM NaCl. Predicted molecular weight based on retention time (RT) from (e) is listed and corresponds to monomeric proteins (red square). Intact mass spectrometry of (**i**) E6AP, (**j**) p53^{core}, or (**k**) 16E6 is shown, with the predicted MW indicated. Quality control testing for MBP-16E6(4C4S) protein used in biochemical studies depicting the (**l**) size exclusion chromatography, (**m**) 1 µg resolved by SDS-PAGE followed by Coomassie Blue staining and (**n**) intact mass spectrometry. MW standards are depicted to the left of the SDS-PAGE gel or above of the SEC A280 trace.

a

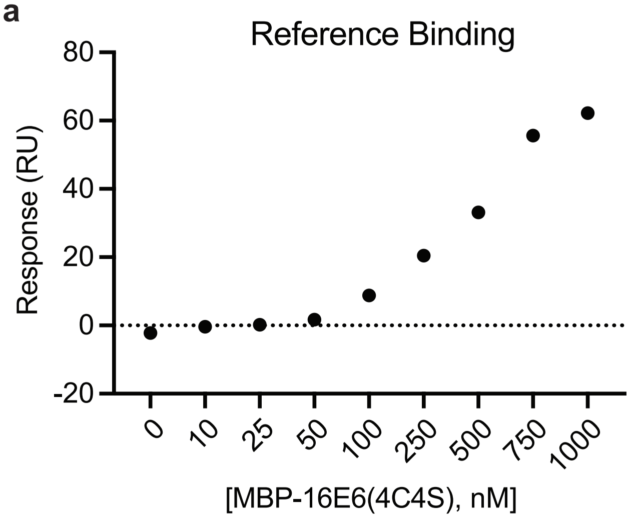

b

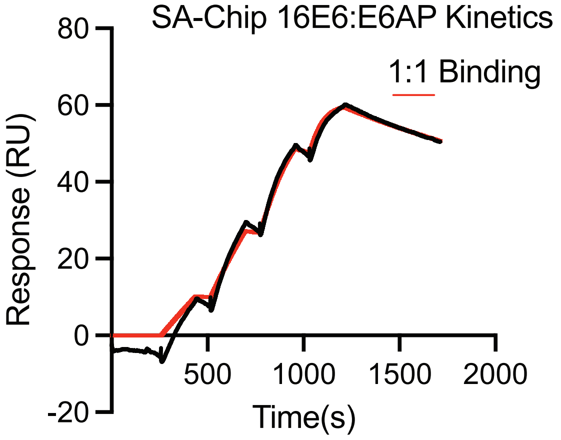

c

Replicate	Ka ($M^{-1}s^{-1}$)	SE (Ka) ±	Kd (s^{-1})	SE (Kd) ±	mean KD (nM)	SE (KD) ±
1	2.67E+06	6.40E+04	5.72E-04	9.90E-06	0.206	0.005
2	1.76E+06	9.40E+03	3.64E-04	2.50E-06		
3	2.19E+06	1.20E+04	4.31E-04	2.80E-06		

Supplementary Figure 4. Control experiments validating parameters for surface plasmon resonance (SPR) kinetic measurements. (**a**) SPR measurement showing nonspecific binding of MBP-16E6(4C4S) to the reference flowcell surface without immobilized E6AP over 180 seconds at 30 µL/min. CAP sensorchip conditioning with the CAPture reagent revealed nonspecific binding above 50 nM. To prevent nonspecific interactions with the sensor chip, all SPR experiments were conducted with analyte concentrations under 50 nM. (**b**) Representative sensorgram of a single cycle kinetics of analysis of MBP-16E6(4C4S) binding to biotinylated E6AP immoblized on a streptavidin SA sensorchip. MBP-16E6(4C4S) and E6AP binding is retained in the absence of DNA oligonucleotides that are present on the CAP sensorchip used in all other SPR measurements. (**c**) Kinetic data derived from three independent single-cycle kinetic experiments assessing the interaction between MBP-16E6(4C4S) and biotinylated E6AP on SA sensorchips using a 1:1 binding model. Abbreviations: RU (response units), K_D (dissociation constant), K_a (association rate), K_d (dissociation rate), and SE (standard error of the mean).

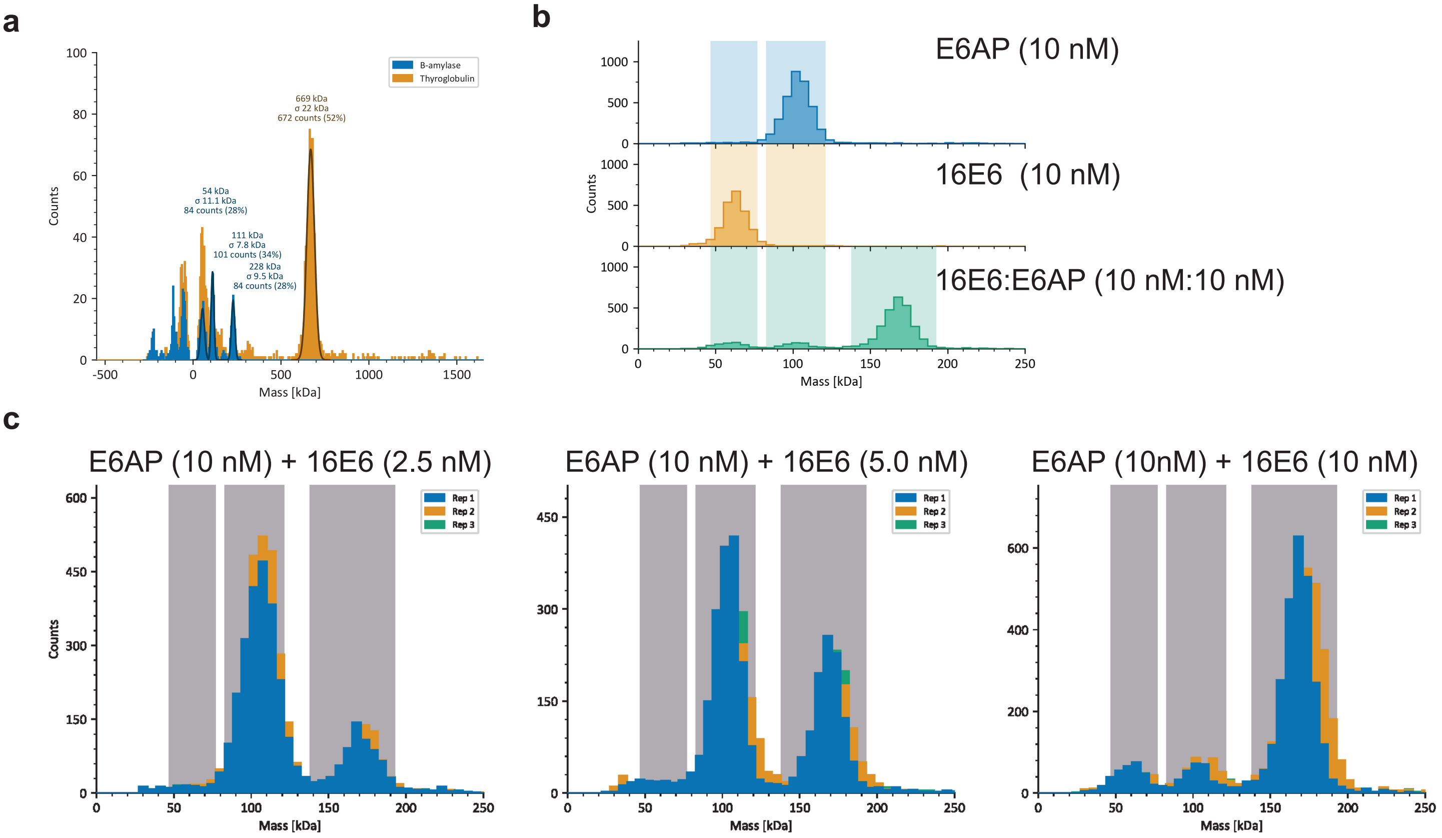

d

	[E6AP] initial	[16E6] initial	E6AP counts	16E6 counts	E6AP:16E6 counts	E6AP adj counts	16E6 adj counts	E6AP:16E6 adj counts	f conversion	[E6AP]eq (nM)	[16E6]eq (nM)	[E6AP:16E6]eq (nM)		KD (nM)
Replicate 1	10	0	3247	66	0									
	0	10	50	2107	0									
	10	2.5	2117	81	666	2067	15	666	3.66E-03	7.56	0.06	2.44		0.20
	10	5	1619	106	1070	1569	40	1070	3.79E-03	5.95	0.95	4.05		1.39
	10	10	298	284	2538	248	218	2538	3.59E-03	0.89	0.89	9.11		0.09
Replicate 2	10	0	2431	57	0									
	0	10	86	65	0									
	10	2.5	2304	82	667	2218	25	667	3.47E-03	7.69	0.19	2.31		0.63
	10	5	1299	64	949	1213	7	949	4.63E-03	5.61	0.61	4.39		0.78
	10	10	410	212	2918	324	155	2918	3.08E-03	1.00	1.00	9.00		0.11
Replicate 3	10	0	2851	47	0									
	0	10	35	1098	0									
	10	2.5	1804	73	489	1769	26	489	4.43E-03	7.83	0.33	2.17		1.21
	10	5	1528	72	958	1493	25	958	4.08E-03	6.09	1.09	3.91		1.70
	10	10	288	209	2480	253	162	2480	3.66E-03	0.93	0.93	9.07		0.09
											**E6AP (nM)**	**16E6 (nM)**	**Mean (KD)**	**SD**
											10	2.5	0.677	0.51
											10	5	1.289	0.47
											10	10	0.097	0.01
												**Average**	**0.688**	0.60

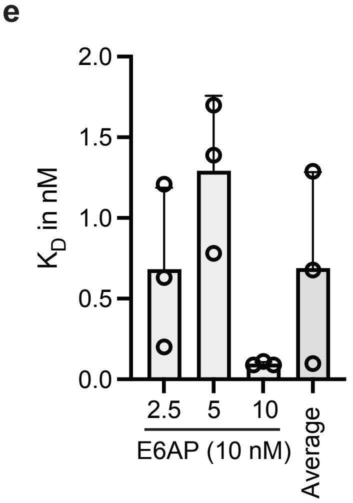

Supplementary Figure 5. (**a**) A calibration curve for mass photometry was plotted using the proteins sweet potato β-amylase (with a molecular weight of ~228 kDa in the tetrameric form, ~111 kDa in the dimeric form, and ~54 kDa in the monomeric form), and thyroglobulin (~660 kDa). (**b**) The mass histograms for MBP-16E6 at a concentration of 10 nM (top), E6AP at 10 nM (middle), and the E6AP:16E6 complex (bottom) (**c**) Mass histograms of E6AP, 16E6, and E6:E6AP complex at tirating concentrations of 16E6. (**d**) Counts obtained from Gaussian fits to the mass histograms. A conversion factor for the conversion of counts to molar concentrations, *f* conversion, is equal to $[E6AP]_{initial}$ / (E6AP counts + E6AP:16E6 counts). Following the mass balance model, the molar concentrations of each species at equilibrium can be calculated; [E6AP]eq = E6AP counts * *f* conversion, [16E6]eq = 16E6 counts * *f* conversion, [E6AP:16E6]eq = E6AP:16E6 counts * *f* conversion. The equilibrium dissociating constant, $K_{D,}$ is calulated by the ratio [E6AP:16E6]eq / ([E6AP]eq * [16E6]eq). (**e**) Graphic comprehensive overview of the K_D values representing the average and standard deviation from three independent trials.

a

```
             HisTag AviTag TEV site           E6AP
E6AP_cryo    ----------------------------MEKLHQCYWKSGEPQSDDIEASRMKRAAAKHL
E6AP_BRET    HHHHHHGLNDIFEAQKIEWHEENLYFQGMEKLHQCYWKSGEPQSDDIEASRMKRAAAKHL

E6AP_cryo    IERYYHQLTEGCGNEACTNEFCASCPTFLRMDNNAAAIKALELYKINAKLCDPHPSKKGA
E6AP_BRET    IERYYHQLTEGCGNEACTNEFCASCPTFLRMDNNAAAIKALELYKINAKLCDPHPSKKGA

E6AP_cryo    SSAYLENSKGAPNNSCSEIKMNKKGARIDFKDVTYLTEEKVYEILELCREREDYSPLIRV
E6AP_BRET    SSAYLENSKGAPNNSCSEIKMNKKGARIDFKDVTYLTEEKVYEILELCREREDYSPLIRV

E6AP_cryo    IGRVFSSAEALVQSFRKVKQHTKEELKSLQAKDEDKDEDEKEKAACSAAAMEEDSEASSS
E6AP_BRET    IGRVFSSAEALVQSFRKVKQHTKEELKSLQAKDEDKDEDEKEKAACSAAAMEEDSEASSS

E6AP_cryo    RIGDSSQGDNNLQKLGPDDVSVDIDAIRRVYTRLLSNEKIETAFLNALVYLSPNVECDLT
E6AP_BRET    RIGDSSQGDNNLQKLGPDDVSVDIDAIRRVYTRLLSNEKIETAFLNALVYLSPNVECDLT

E6AP_cryo    YHNVYSRDPNYLNLFIIVMENRNLHSPEYLEMALPLFCKAMSKLPLAAQGKLIRLWSKYN
E6AP_BRET    YHNVYSRDPNYLNLFIIVMENRNLHSPEYLEMALPLFCKAMSKLPLAAQGKLIRLWSKYN

E6AP_cryo    ADQIRRMMETFQQLITYKVISNEFNSRNLVNDDDAIVAASKCLKMVYYANVVGGEVDTNH
E6AP_BRET    ADQIRRMMETFQQLITYKVISNEFNSRNLVNDDDAIVAASKCLKMVYYANVVGGEVDTNH

E6AP_cryo    NEEDDEEPIPESSELTLQELLGEERRNKKGPRVDPLETELGVKTLDCRKPLIPFEEFINE
E6AP_BRET    NEEDDEEPIPESSELTLQELLGEERRNKKGPRVDPLETELGVKTLDCRKPLIPFEEFINE

E6AP_cryo    PLNEVLEMDKDYTFFKVETENKFSFMTCPFILNAVTKNLGLYYDNRIRMYSERRITVLYS
E6AP_BRET    PLNEVLEMDKDYTFFKVETENKFSFMTCPFILNAVTKNLGLYYDNRIRMYSERRITVLYS

E6AP_cryo    LVQGQQLNPYLRLKVRRDHIIDDALVRLEMIAMENPADLKKQLYVEFEGEQGVDEGGVSK
E6AP_BRET    LVQGQQLNPYLRLKVRRDHIIDDALVRLEMIAMENPADLKKQLYVEFEGEQGVDEGGVSK

E6AP_cryo    EFFQLVVEEIFNPDIGMFTYDESTKLFWFNPSSFETEGQFTLIGIVLGLAIYNNCILDVH
E6AP_BRET    EFFQLVVEEIFNPDIGMFTYDESTKLFWFNPSSFETEGQFTLIGIVLGLAIYNNCILDVH

E6AP_cryo    FPMVVYRKLMGKKGTFRDLGDSHPVLYQSLKDLLEYEGNVEDDMMITFQISQTDLFGNPM
E6AP_BRET    FPMVVYRKLMGKKGTFRDLGDSHPVLYQSLKDLLEYEGNVEDDMMITFQISQTDLFGNPM

E6AP_cryo    MYDLKENGDKIPITNENRKEFVNLYSDYILNKSVEKQFKAFRRGFHMVTNESPLKYLFRP
E6AP_BRET    MYDLKENGDKIPITNENRKEFVNLYSDYILNKSVEKQFKAFRRGFHMVTNESPLKYLFRP

E6AP_cryo    EEIELLICGSRNLDFQALEETTEYDGGYTRDSVLIREFWEIVHSFTDEQKRLFLQFTTGT
E6AP_BRET    EEIELLICGSRNLDFQALEETTEYDGGYTRDSVLIREFWEIVHSFTDEQKRLFLQFTTGT

E6AP_cryo    DRAPVGGLGKLKMIIAKNGPDTERLPTSHTCFNVLLLPEYSSKEKLKERLLKAITYAKGF
E6AP_BRET    DRAPVGGLGKLKMIIAKNGPDTERLPTSHTCFNVLLLPEYSSKEKLKERLLKAITYAKGF
             TEV site HisTag AviTag     Nanoluciferase
E6AP_cryo    GMLENLYFQGHHHHHHGLNDIFEAQKI--EWHE*--------------------------
E6AP_BRET    GMLVSL----------GSSGVFTLEDFVGDWRQTAGYNLDQVLEQGGVSSLFQNLGVSVT
                             Linker^
E6AP_cryo    ------------------------------------------------------------
E6AP_BRET    PIQRIVLSGENGLKIDIHVIIPYEGLSGDQMGQIEKIFKVVYPVDDHHFKVILHYGTLVI

E6AP_cryo    ------------------------------------------------------------
E6AP_BRET    DGVTPNMIDYFGRPYEGIAVFDGKKITVTGTLWNGNKIIDERLINPDGSLLFRVTINGVT

E6AP_cryo    ----------
E6AP_BRET    GWRLCERILA
```

```
              HisTag                   MBPTag
4C4S_biochem  MAHHHHHHPMKIEEGKLVIWINGDKGYNGLAEVGKKFEKDTGIKVTVEHPDKLEEKFPQV
4C4S_BRET     MAHHHHHHPMKIEEGKLVIWINGDKGYNGLAEVGKKFEKDTGIKVTVEHPDKLEEKFPQV

4C4S_biochem  AATGDGPDIIFWAHDRFGGYAQSGLLAEITPDKAFQDKLYPFTWDAVRYNGKLIAYPIAV
4C4S_BRET     AATGDGPDIIFWAHDRFGGYAQSGLLAEITPDKAFQDKLYPFTWDAVRYNGKLIAYPIAV

4C4S_biochem  EALSLIYNKDLLPNPPKTWEEIPALDKELKAKGKSALMFNLQEPYFTWPLIAADGGYAFK
4C4S_BRET     EALSLIYNKDLLPNPPKTWEEIPALDKELKAKGKSALMFNLQEPYFTWPLIAADGGYAFK

4C4S_biochem  YENGKYDIKDVGVDNAGAKAGLTFLVDLIKNKHMNADTDYSIAEAAFNKGETAMTINGPW
4C4S_BRET     YENGKYDIKDVGVDNAGAKAGLTFLVDLIKNKHMNADTDYSIAEAAFNKGETAMTINGPW

4C4S_biochem  AWSNIDTSKVNYGVTVLPTFKGQPSKPFVGVLSAGINAASPNKELAKEFLENYLLTDEGL
4C4S_BRET     AWSNIDTSKVNYGVTVLPTFKGQPSKPFVGVLSAGINAASPNKELAKEFLENYLLTDEGL

4C4S_biochem  EAVNKDKPLGAVALKSYEEELAKDPRIAATMENAQKGEIMPNIPQMSAFWYAVRTAVINA
4C4S_BRET     EAVNKDKPLGAVALKSYEEELAKDPRIAATMENAQKGEIMPNIPQMSAFWYAVRTAVINA
                                Linker  TEV site  HPV16 E6 4C4S
4C4S_biochem  ASGRQTVDEALKDAQTNSSSNNNNNNNNNNPMSENLYFQGAMFQDPQERPRKLPQLCTEL
4C4S_BRET     ASGRQTVDEALKDAQTNSSSNNNNNNNNNNPMSENLYFQGAMFQDPQERPRKLPQLCTEL

4C4S_biochem  QTTIHDIILECVYCKQQLLRREVYDFAFRDLCIVYRDGNPYAVCDKCLKFYSKISEYRHY
4C4S_BRET     QTTIHDIILECVYCKQQLLRREVYDFAFRDLCIVYRDGNPYAVCDKCLKFYSKISEYRHY

4C4S_biochem  SYSLYGTTLEQQYNKPLSDLLIRCINCQKPLSPEEKQRHLDKKQRFHNIRGRWTGRCMSC
4C4S_BRET     SYSLYGTTLEQQYNKPLSDLLIRCINCQKPLSPEEKQRHLDKKQRFHNIRGRWTGRCMSC
                            Linker       HaloTag
4C4S_biochem  SRSSRTRRETQL------------------------------------------------
4C4S_BRET     SRSSRTRRETQLVSLEPTTDNDGSEIGTGFPFDPHYVEVLGERMHYVDVGPRDGTPVLFL

4C4S_biochem  ------------------------------------------------------------
4C4S_BRET     HGNPTSSYVWRNIIPHVAPTHRCIAPDLIGMGKSDKPDLGYFFDDHVRFMDAFIEALGLE

4C4S_biochem  ------------------------------------------------------------
4C4S_BRET     EVVLVIHDWGSALGFHWAKRNPERVKGIAFMEFIRPIPTWDEWPEFARETFQAFRTTDVG

4C4S_biochem  ------------------------------------------------------------
4C4S_BRET     RKLIIDQNVFIEGTLPMGVVRPLTEVEMDHYREPFLNPVDREPLWRFPNELPIAGEPANI

4C4S_biochem  ------------------------------------------------------------
4C4S_BRET     VALVEEYMDWLHQSPVPKLLFWGTPGVLIPPAEAARLAKSLPNCKAVDIGPGLNLLQEDN

4C4S_biochem  -------------------
4C4S_BRET     PDLIGSEIARWLSTLEISG
```

Supplementary Figure 6a. Sequence alignment of the primary amino acid sequences of E6AP and 16E6. (**a**) The sequences utilized for NanoBRET assays are labeled as E6AP_BRET and 16E6_BRET, whereas those used for CryoEM and biochemical experiments are designated as E6AP_cryo and 16E6_biochem, respectively.

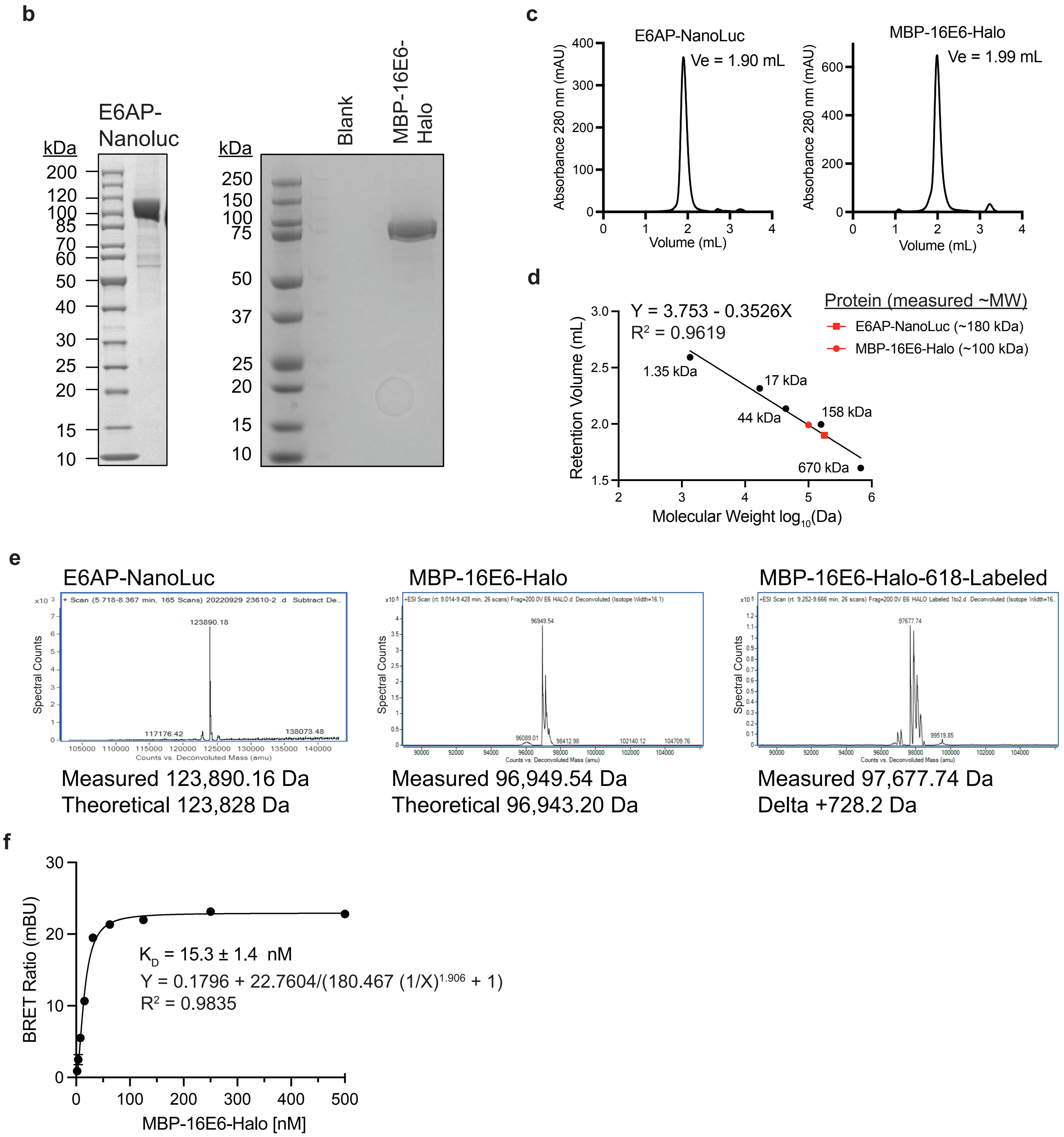

Supplementary Figure 6b. Quality control of NanoBRET proteins and affinity between MBP-16E6-Halo and E6AP-Nanoluc. (**b**) The indicated E6AP-Nanoluc and 16E6-HALO proteins (2 µg) were resolved by SDS-PAGE and visualized with Instant Blue stain. (**c**) NanoBRET proteins were subjected to analytical size exclusion chromatography using a Superdex200 5/150 column, indicating Retention Volume (Ve). (**d**) A gel filtration standard (Bio-Rad, Cat no. 1511901; black circles) was used, and peak retention volumes were plotted against the molecular weights of the respective standards on the same column. Using linear regression, a calibration curve was generated (black line), and molecular weights of the NanoBRET proteins were extrapolated (red square and circle). (**e**) LC-MS deconvoluted intact mass spectras of E6AP-Nanoluc, MBP-16E6-Halo, and MBP-16E6-Halo after Ligand618 labeling. (**f**) In-solution NanoBRET proximity assay measuring binding events of E6AP-NanoLuc titrated against 618-labeled MBP-16E6-Halo revealed a K_D of 15.3 nM. The K_D was calculated using the law of mass action model, assuming a fractional occupancy of 0.5 when the ligand concentration is equal to K_D. Value represents the mean ± 95% confidence interval of the fit where Y=Bottom + (Top-Bottom)/(1 + (EC50/X)^HillSlope).

a

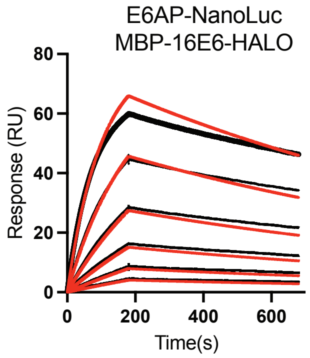

b

Replicate	Ka ($M^{-1}s^{-1}$)	SE (Ka) ±	Kd (s^{-1})	SE (Kd) ±	mean K_D (nM)	SE (KD) ±
1	1.99E+05	1.30E+02	7.04E-04	1.20E-06	3.748	0.108
2	1.87E+05	1.50E+02	7.23E-04	1.60E-06		
3	1.93E+05	1.40E+02	7.39E-04	1.40E-06		

Supplementary Figure 7. Evaluation of binding affinity between E6AP-NanoLuc and MBP-16E6-Halo using Surface Plasmon Resonance (SPR). (**a**) SPR sensograms depicting increasing concentrations of 16E6-Halo through five 2-fold serial dilutions, starting from 50 nM, interacting with immobilized biotinylated E6AP-NanoLuc. The presented plot is representative of three independent experimental replicates. Displayed are the measured binding responses (in black) alongside curve fits based on a 1:1 binding model (in red). (**b**) Kinetics table detailing measurements from three experimental replicates fitted to a 1:1 binding model. RU, response units; K_D, dissociation constant; K_a, association rate; K_d, dissociation rate; SE, standard error of mean.

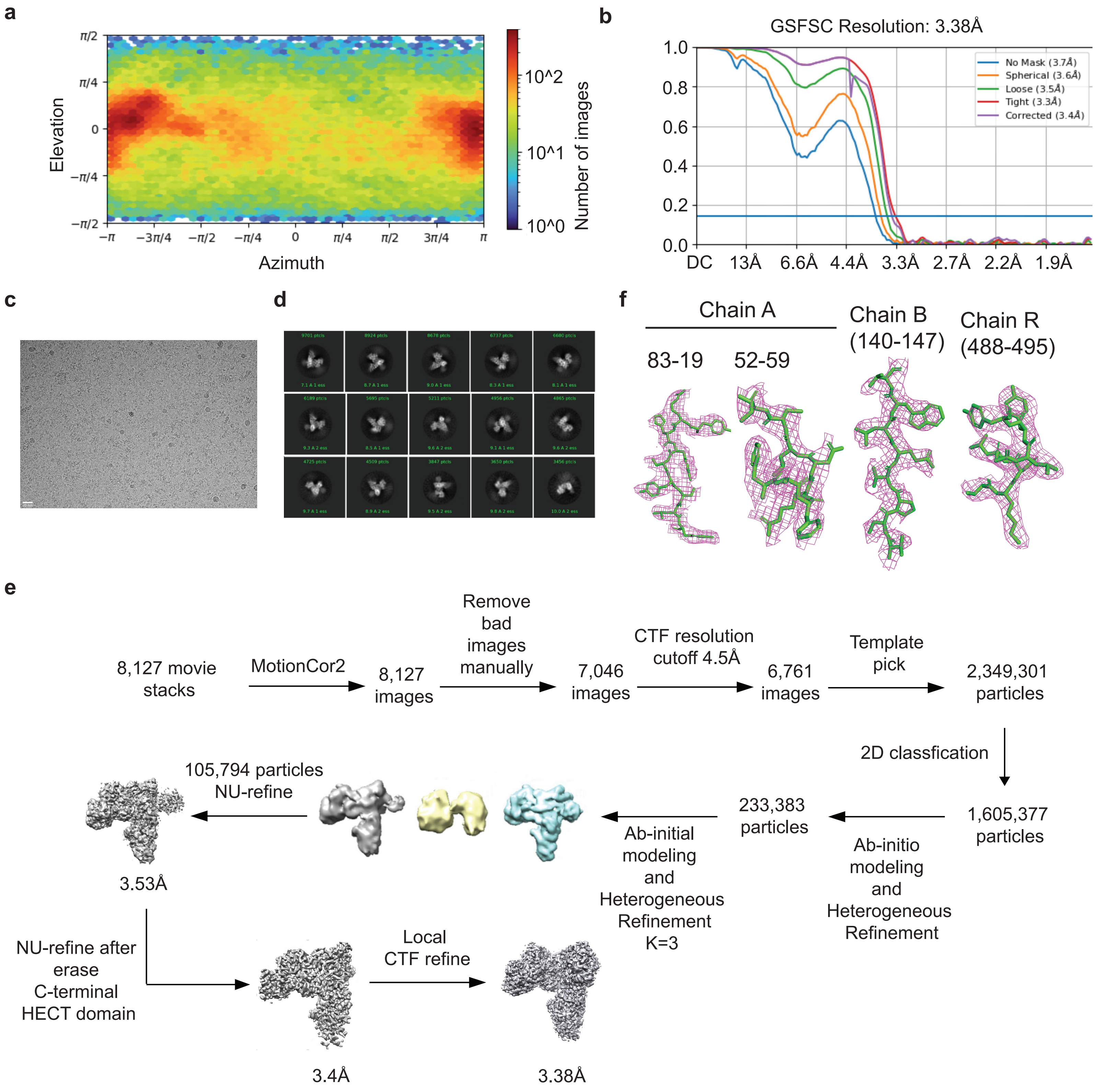

Supplementary Figure 8. CryoEM data processing of the HPV16 E6, E6AP and p53 ternary complex. (**a**) Angular distribution of particle set for the final reconstruction using cryoSPARC. (**b**) Gold-standard Fourier shell correlation for the reconstructed map. The resolution was estimated to be 3.4 Å based on the FSC = 0.143 criterion. (**c**) Representative micrograph of the complex formed by E6AP, MBP-16E6(4C4S), and the p53^{core} domain at a concentration of 2.15 mg/mL. The scale bar corresponds to 20 nanometers. (**d**) Representative 2D classes calculated from extracted particles. (**e**) Flowchart of image processing for the HPV16 E6, E6AP, and p53 ternary complex. Mask generated from HECT-erased volume was applied at the final non-uniform refinement step. (**f**) CryoEM density map is shown as magenta mesh and the atomic model is represented by green sticks. Fitting of the model to the density map of selected regions from Chain A (16E6), Chain B (p53), and Chain R (E6AP) are shown, respectively.

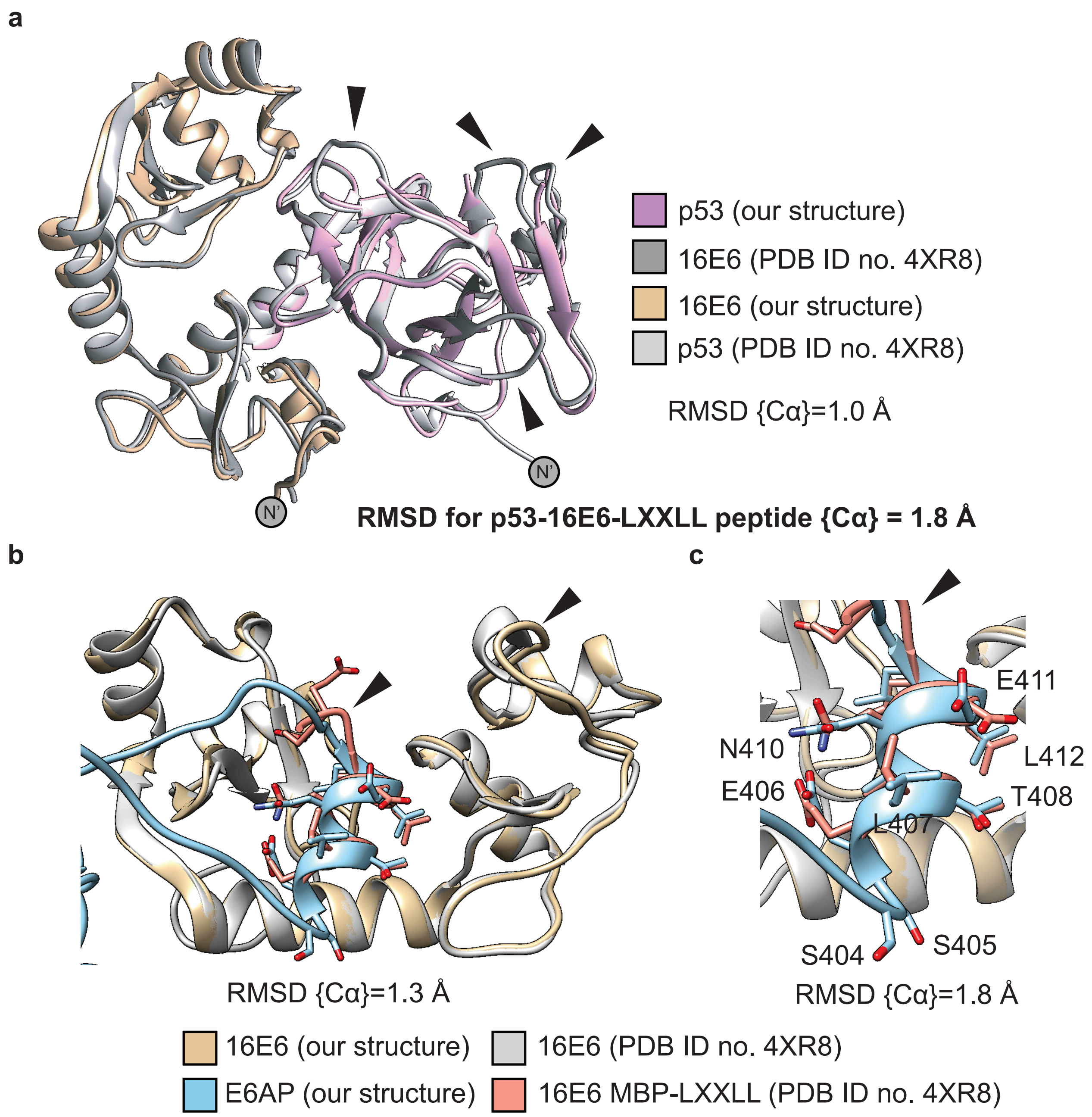

Supplementary Figure 9. Superimposition of 16E6, E6AP, and p53^{core} onto previous structures reveals a high degree of similarity. Individual chains from PDB ID no. 4XR8 were superimposed onto the 16E6, E6AP, and p53^{core} CryoEM ternary complex (blue, purple, tan) for (**a**) 16E6 and p53, (**b**) 16E6 and MBP-LXXLL, and (**c**) a zoomed-in insert highlighting the side chains of the LXXLL peptide of full-length E6AP (blue, ribbon and stick representation) or the MBP-LXXLL (salmon, ribbon and stick representation; ELTLQELLGEER). The MBP portion of the MBP-LXXLL peptide was hidden for clarity. Black arrows indicate areas with noticeable differences in backbone conformation or missing loops. The RMSD between 138 pruned atom pairs for E6AP is 0.58Å and across all 140 pairs is 0.63Å, while the RMSD between 172 pruned atom pairs of p53 is 0.66Å and across all 176 atom pairs is 0.93Å.

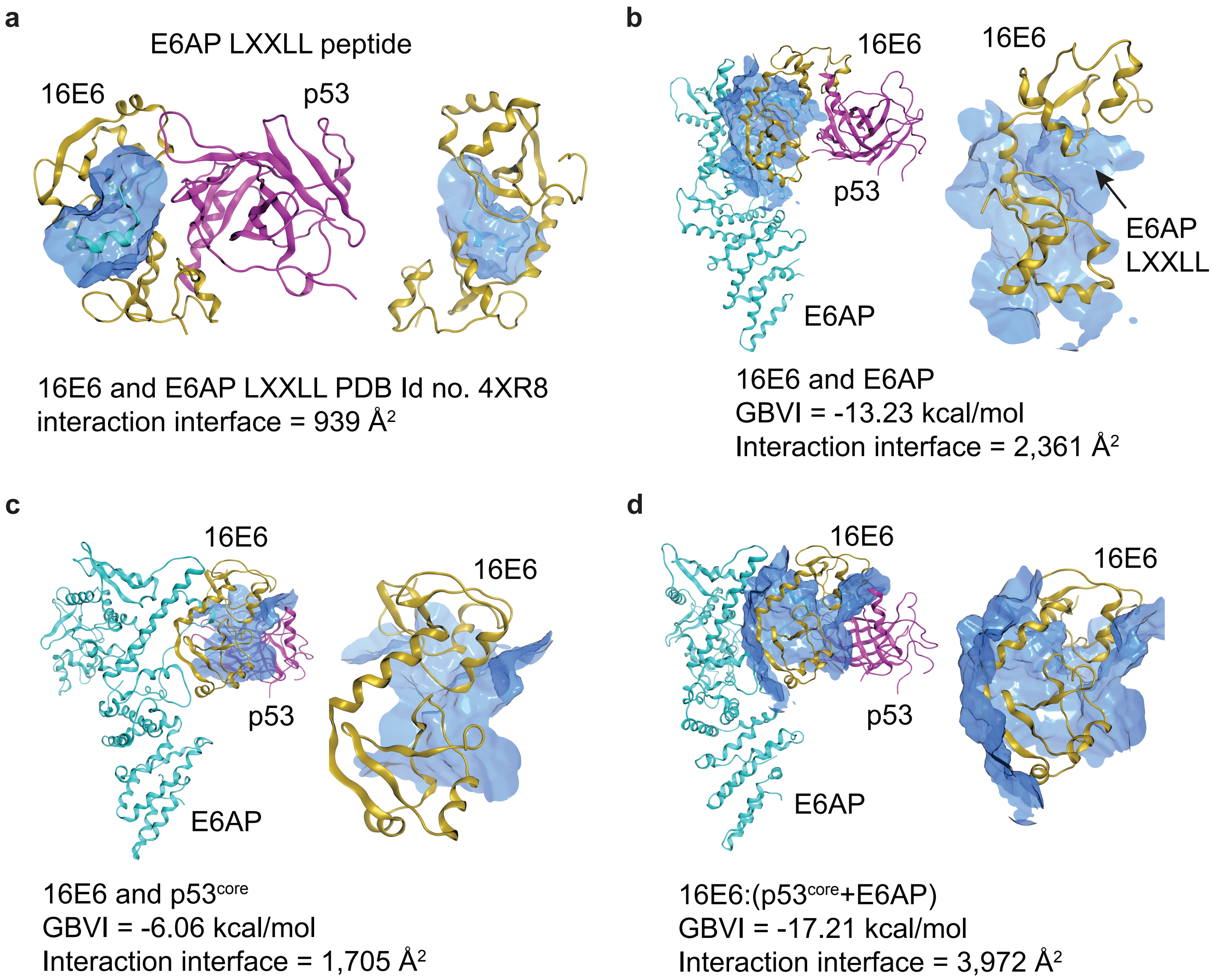

Supplementary Figure 10. Interaction interfaces between protein subunits in the 16E6, E6AP and p53^{core} ternary complex. (**a**) The HPV 16E6 (gold), p53^{core} domain (magenta) and the LXXLL E6AP peptide (blue) complex (PDB ID no. 4XR8), all in ribbon are depicted with the 16E6:LXXLL peptide:protein interaction interface (translucent blue surface) displayed. The HPV 16E6 (gold), p53^{core} domain (magenta) and the full length E6AP protein (blue) interaction interfaces are depicted as a blue translucent surface between 16E6 and (**b**) E6AP, (**c**) p53^{core}, or (**d**) E6AP and p53^{core} each with zoomed-in view of the interface displayed with 16E6 (gold, ribbon representation). Interaction surface area was determined using the protein contact utility in MOE and is listed below its respective panel. Structure was prepared using the MOE structure preparation utility to protonate and solvate the system prior to calculating the GBVI and surface area.

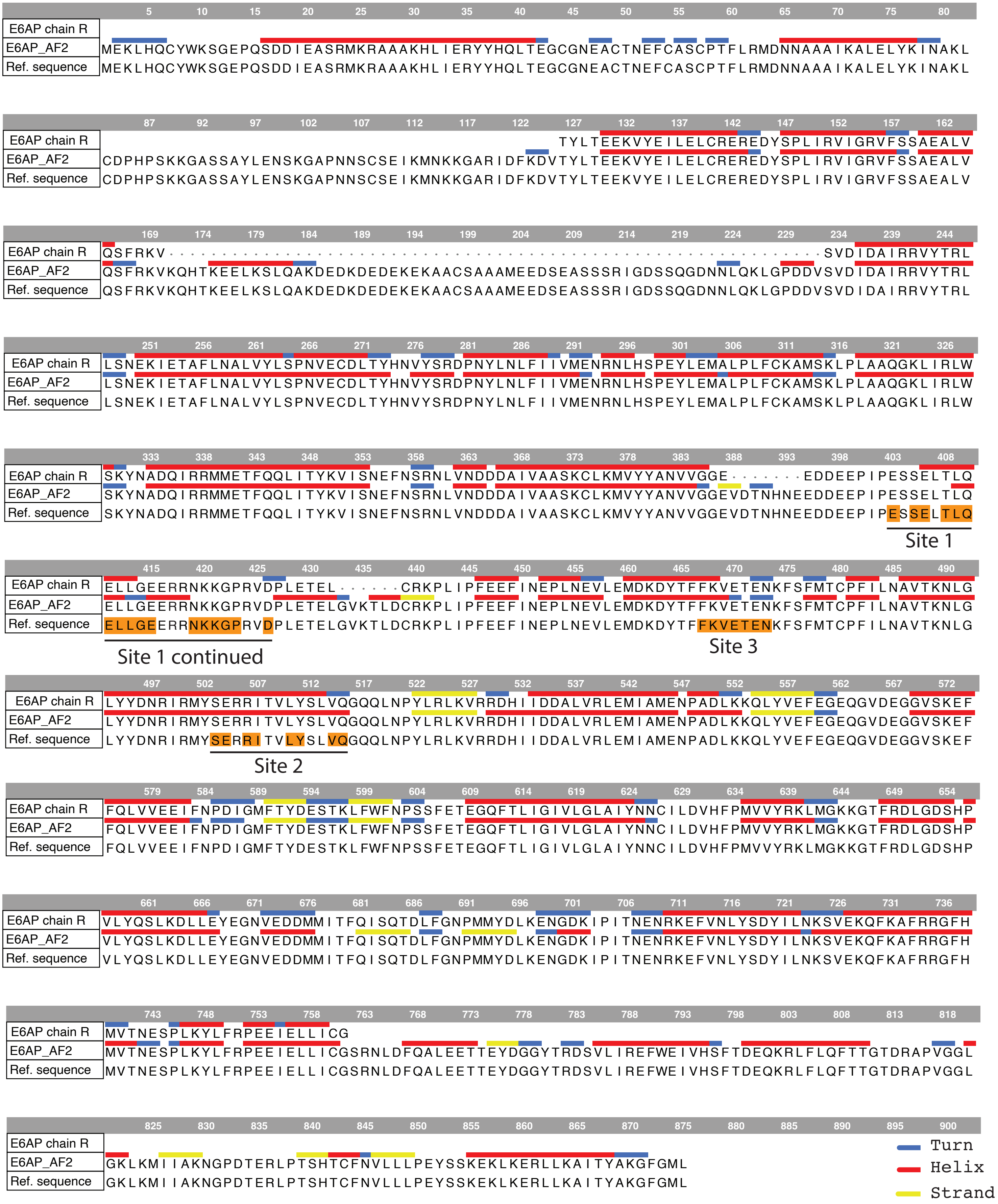

Supplementary Figure 11. Alignment of E6AP sequence and secondary structure with key interacting partners. The primary sequence of E6AP (UniProt accession no. Q05086) was aligned with our cryoEM structure (chain R) or the AlphaFold2 structure (Ref # AF-Q05086-F1), and secondary structure elements are displayed above the respective sequence when available. Alignment and secondary structure elements were generated using MOE (Chemical Computing Group, version 2020.0901). Site1, site 2, and site 3 interaction interfaces are colored in orange.

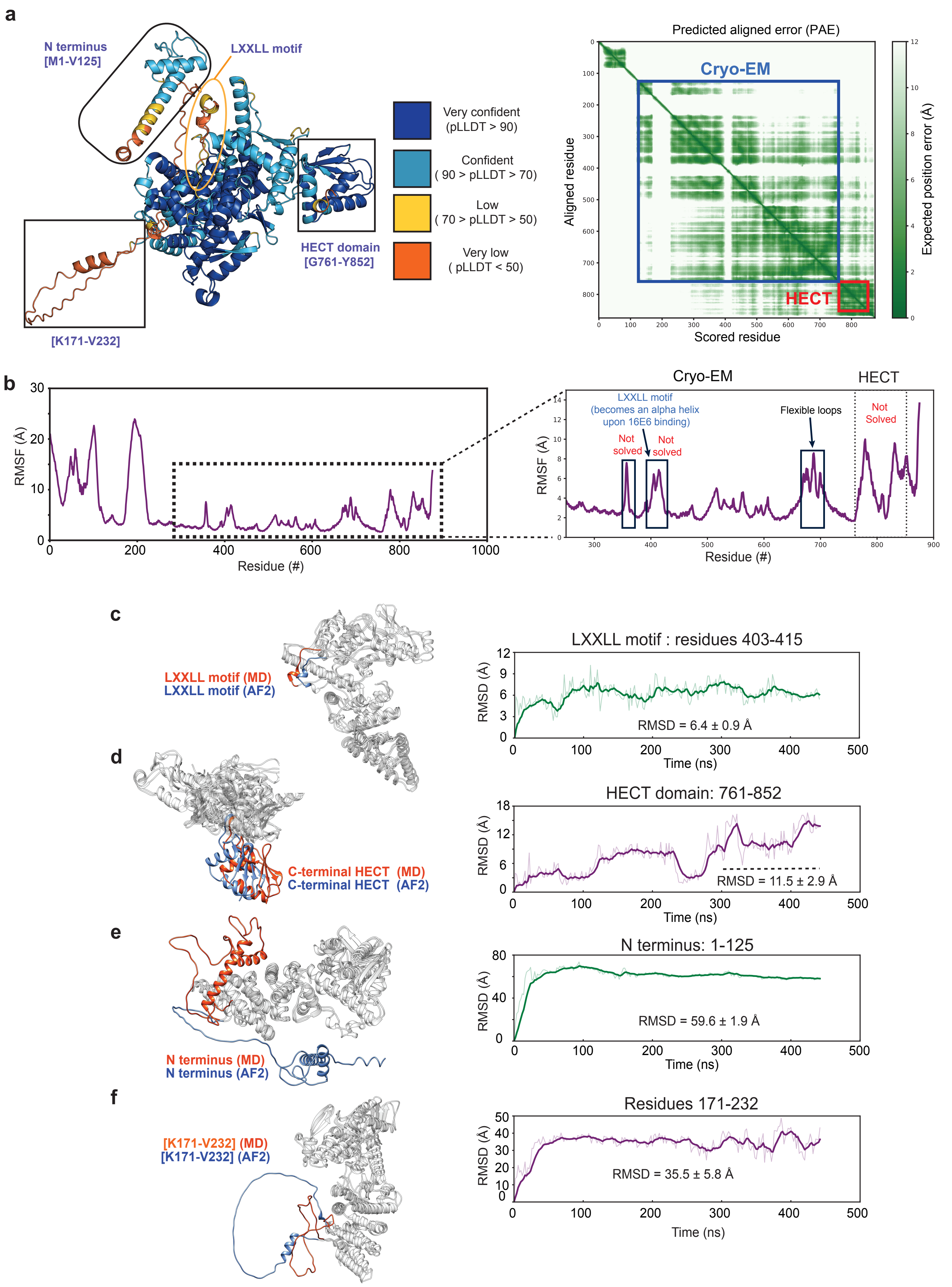

Supplementary Figure 12. Structural analysis of the AlphaFold2 (AF2) predicted E6AP structure by molecular dynamics simulation (MD). (**a**) Top: the AF2 predicted model of E6AP [AF-Q05086-F1-model_v4] with its associated (left) pLDDT and (right) predicted aligned error (PAE) score which quantifies model confidence. (**b**) The root mean square fluctuation (RMSF) analysis of the AlphaFold2 model of the apo-E6AP structure obtained after a ~400 ns MD simulation. (**c**) Left: comparison between the AF2 and the refined structure obtained after 400 ns MD simulation for the LXXLL motif. Right: the trajectory analysis of the root mean square deviation (RMSD) of the LXXLL motif for Cα atoms. (**d**) Left: comparison between the AF2 and the refined structure obtained after 400 ns MD simulation for the C-terminal HECT domain [residues G761-Y852]. Right: the trajectory analysis of RMSD of the C-terminal HECT domain for Cα atoms. (**e**) Left: comparison between the AF2 and the refined structure obtained after 400 ns MD simulation for the N-terminal domain [residues M1-V125]. Right: the trajectory analysis of RMSD of the N-terminal domain for Cα atoms. (**f**) Left: comparison between the AF2 and the refined structure obtained after 400 ns MD simulation for the residues: K171-V232. Right: the trajectory analysis of RMSD for Cα atoms. To determine the RMSD for individual domains, we aligned the E6AP protein from the MD simulation to the AF2 reference structure using the stable Cα atoms of residues [126-170, 233-387, 395-432, and 439-758]. These residues showed stability, with an RMSD of 3.1 ± 0.3 Å.

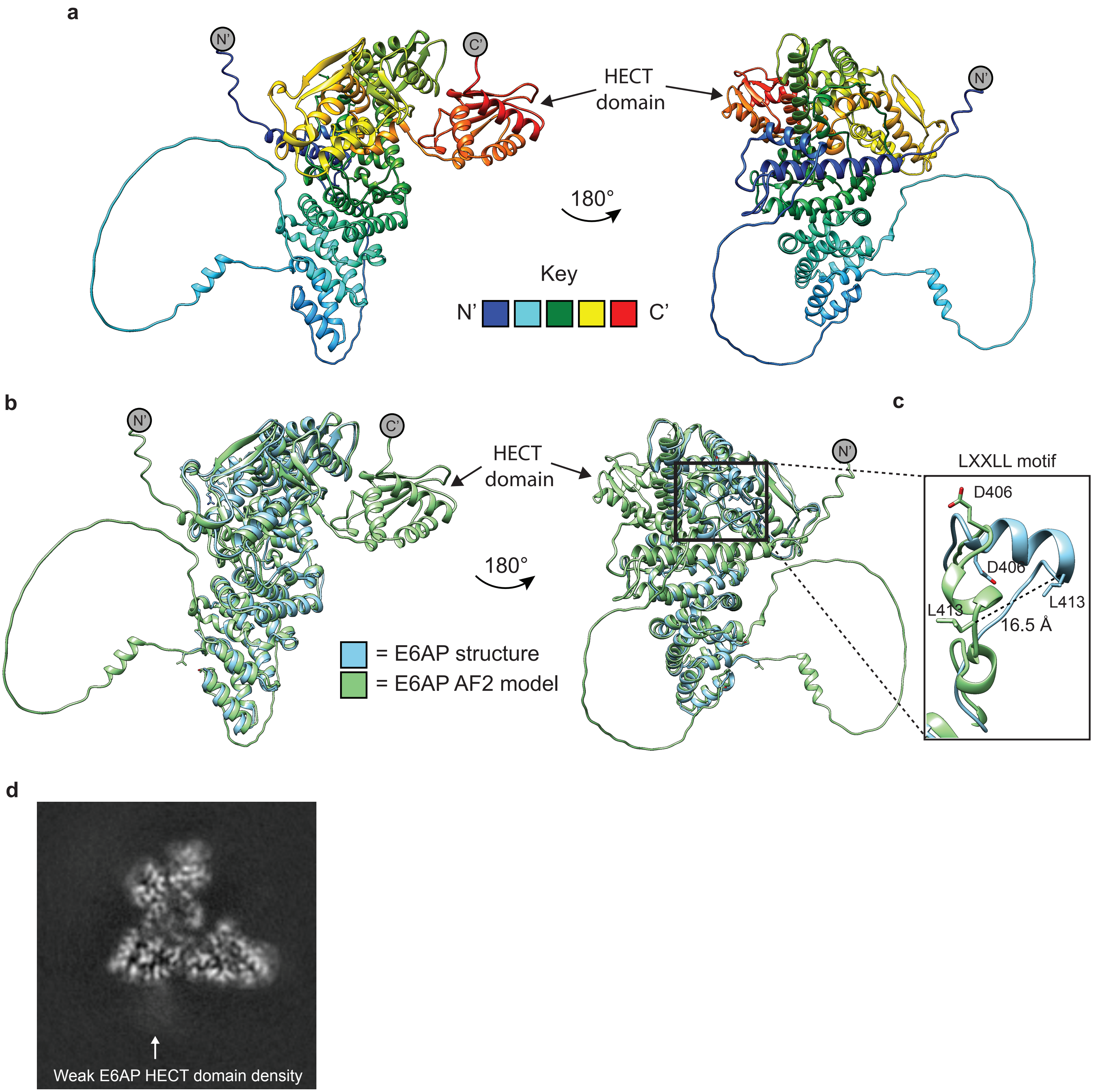

Supplementary Figure 13. Alphafold 2 (AF2) predicted structure of E6AP is consistent with experimentally derived structures. (**a**) AF2 predicted structure of E6AP with rainbow coloration to highlight residue positions (N-terminal, blue; C-terminal, red) is shown with a 180° rotation. HECT domain is shown in red-orange coloration. (**b**) Superimposition of the cryoEM structure of E6AP (blue, ribbon representation) onto the AF2 E6AP structure (green, ribbon representation) is shown with a 180° rotation. (**c**) Zoomed-in view of (b, black dashed-box) highlighting a large conformational change (16.5 Å, backbone carbon of L415) between the unbound (AF2 model, green) and the cryoEM structure (blue). The root mean square deviation (RMSD) between 462 pruned atom pairs is 1.1 Å, and across all 561 pairs, it is 2.8 Å. The AF2 structure version used is "AF-Q05086-F1-model_v2.pdb". (**d**) Map projection of the MBP-16E6, E6AP, and p53 core domains, with a white arrow indicating the low-density region corresponding to the predicted HECT domain of E6AP.

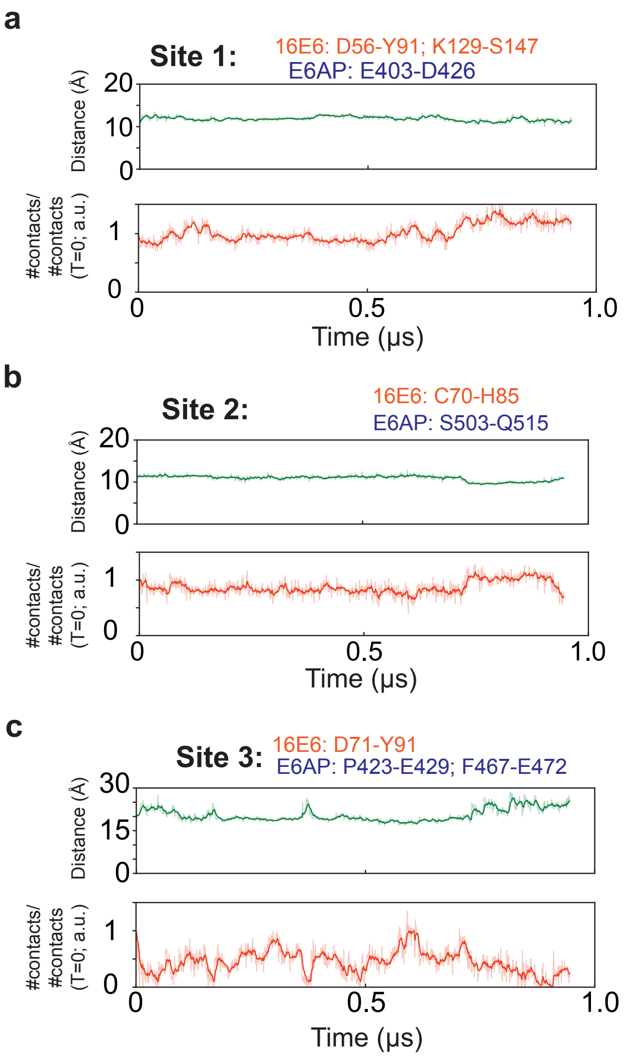

Supplementary Figure 14. Data for the stability analysis and contact interactions between E6AP and 16E6 at (**a**) Site 1, (**b**) Site 2, and (**c**) Site 3 interfaces obtained from the second 1 μs replicate from Figure 3b-d.

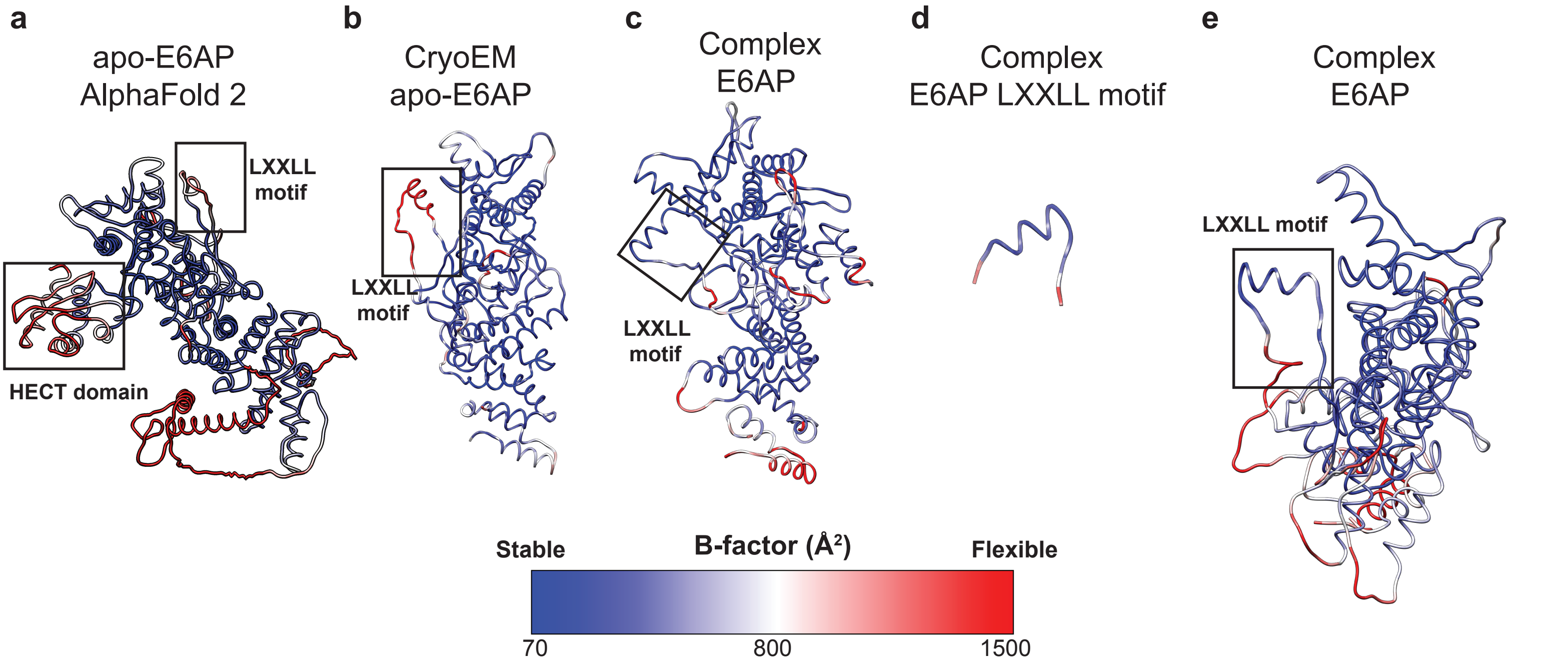

Supplementary Figure 15. The analysis of the E6AP protein flexibility in different states. The averaged structures of E6AP, with the B-factor values represented by a color gradient ranging from blue (representing regions of high stability) to red (representing regions of high flexibility). These values were obtained by calculating the root mean squared fluctuation (RMSF) and provide insights into the dynamic behavior of the E6AP and its LXXLL motif peptide. Initial configurations for the molecular dynamics simulation are as follows: (**a**) apo state of the E6AP protein as predicted by AF2, (**b**) apo state of E6AP after removing p53 and 16E6 from our cryo-EM complex, (**c**) binary complex of 16E6-bound E6AP, derived from the cryoEM structure with p53 removed, (**d**) ternary complex of p53-16E6-E6AP-LXXLL motif, using only the mentioned section and omitting the rest of the E6AP construct from the cryoEM, and (**e**) ternary complex of p53-16E6-E6AP as observed in our cryoEM.

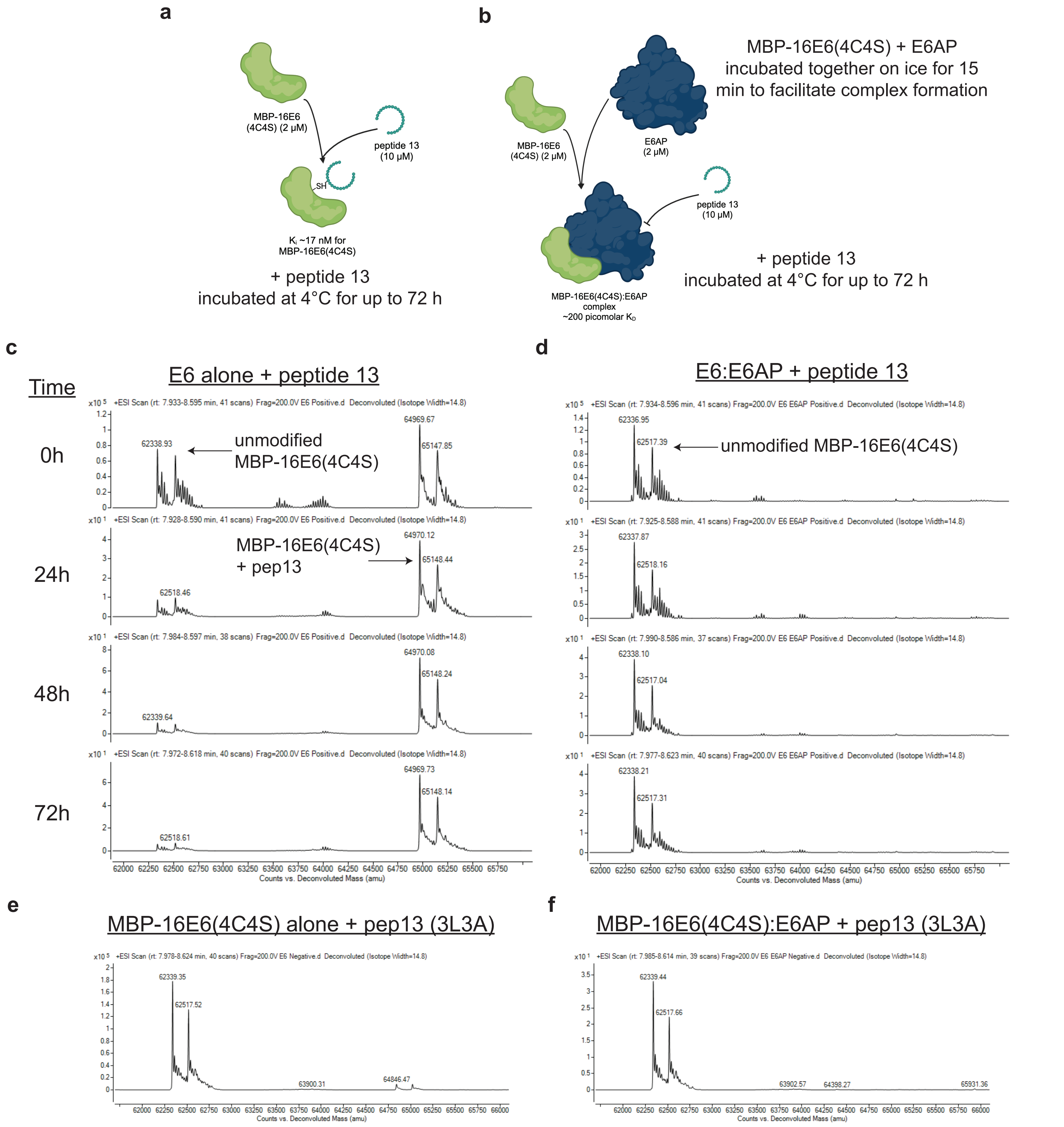

Supplementary Figure 16. Interaction Analysis of Pep13 with MBP-16E6(4C4S) in the Absence and Presence of E6AP. (**a**) Schematic depicting the conjugation of pep13 to MBP-16E6(4C4S). (**b**) Diagram illustrating the inhibition of pep13 binding by E6AP, created with BioRender.com. (**c**) Time-course of pep13 conjugation to MBP-16E6(4C4S) at 0, 24, 48, and 72 hours, demonstrating complete conjugation. The 2,630.14 Da mass of pep13 corresponds to the mass shift observed in MBP-16E6(4C4S), indicating a single conjugation event. (**d**) Equimolar E6AP binding to MBP-16E6(4C4S) blocks the pep13 binding site, preventing conjugation at all observed time-points. (**e**) Control peptide 3L3A, known to not conjugate with MBP-16E6(4C4S), shows no conjugation after 72 hours. (**f**) 3L3A does not conjugate to the MBP-16E6(4C4S) and E6AP complex either. Data reflect results from two independent experiments.

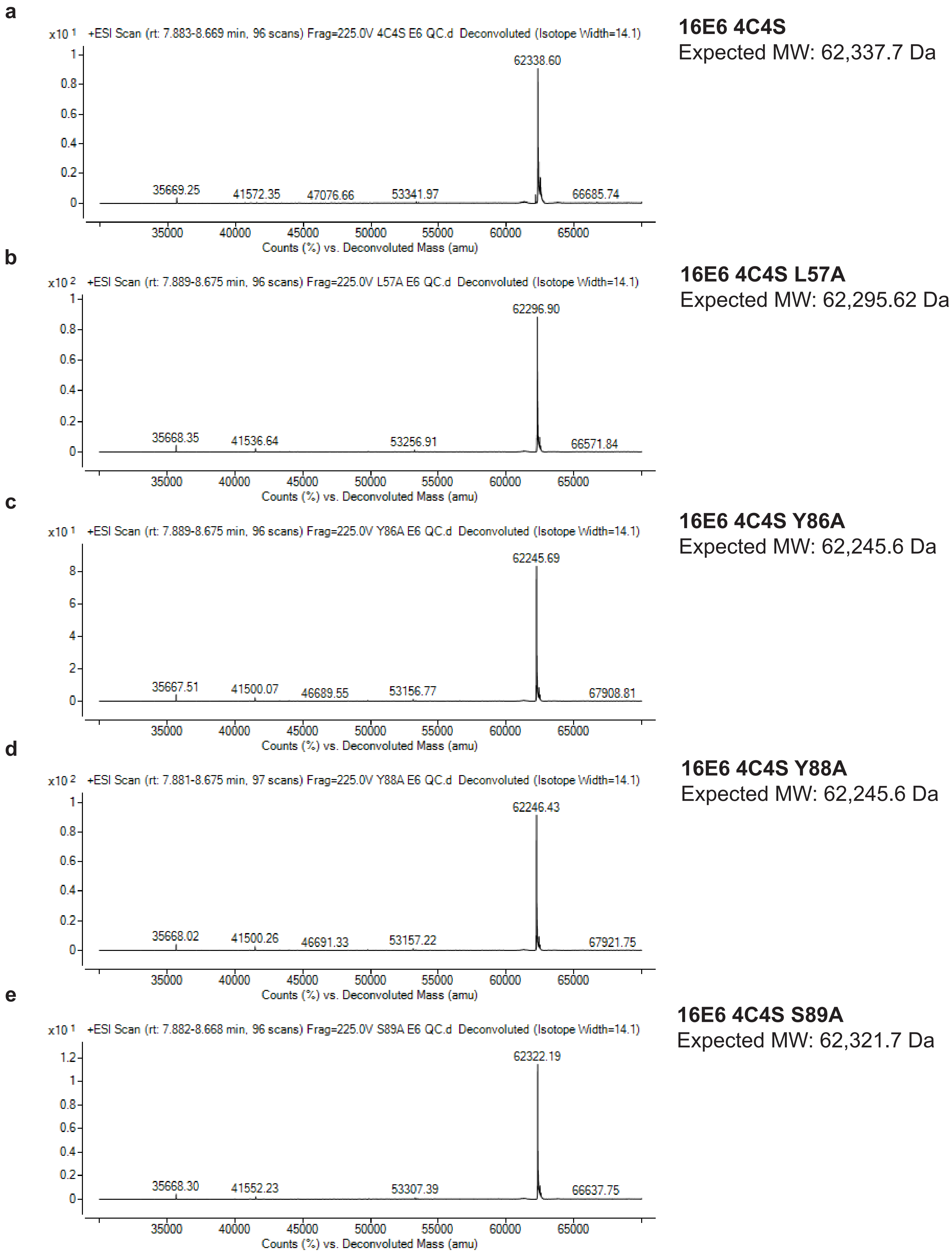

Supplementary Figure 17a. LC-MS characterization of recombinant 16E6(4C4S) proteins (**a**) 4C4S, (**b**) 4C4S L57A, (**c**) 4C4S Y86A, (**d**) 4C4S Y88A, (**e**) 4C4S S89A. Expected molecular weights are calculated with the removal of the translational N-terminal methionine.

f

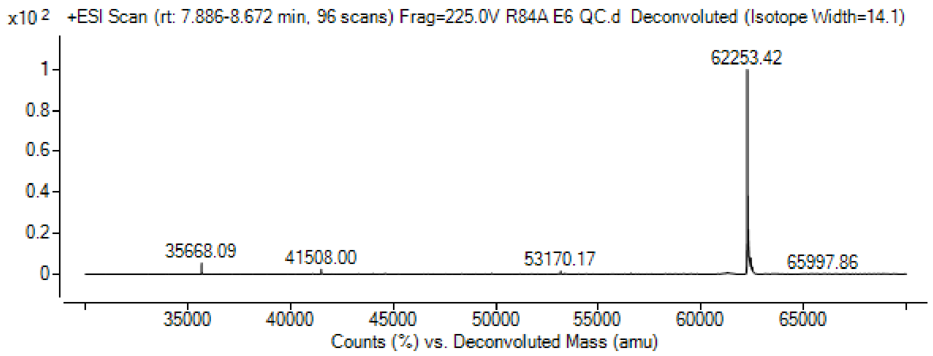

16E6 R84A
Expected MW: 62,252.59 Da

g

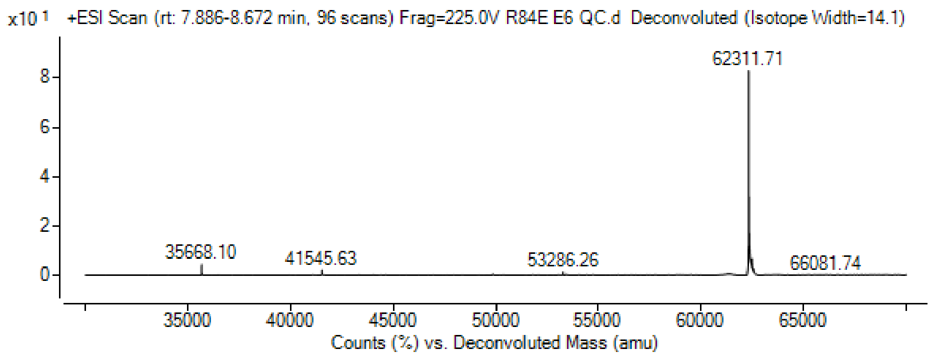

16E6 4C4S R84E
Expected MW: 62,310.63 Da

h

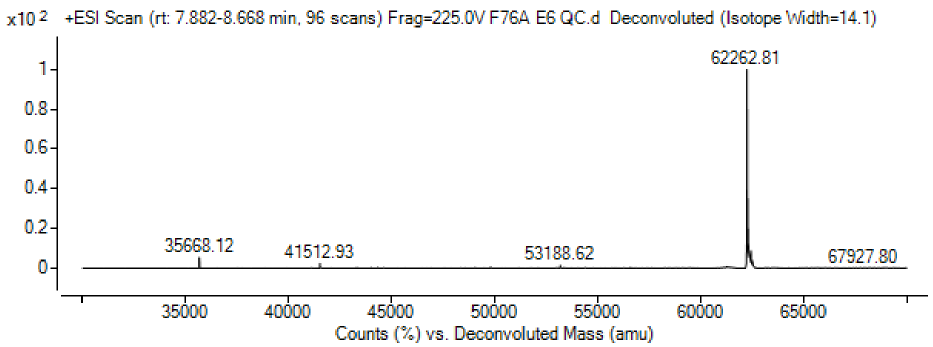

16E6 4C4S F76A
Expected MW: 62,261.60 Da

i

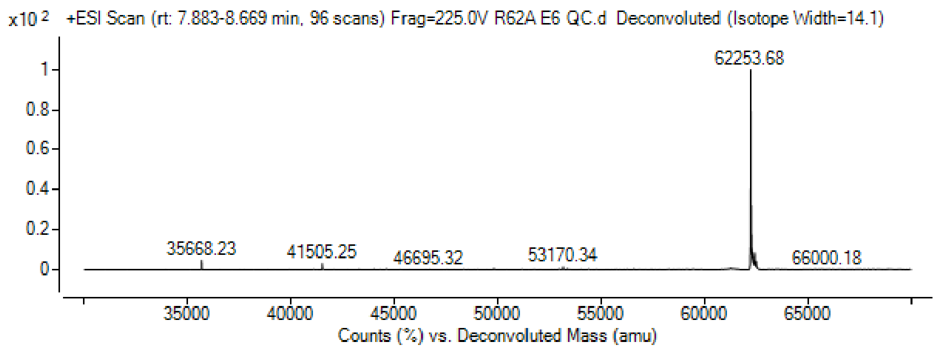

16E6 4C4S R62A
Expected MW: 62,252.59 Da

Supplementary Figure 17b. LC-MS characterization of recombinant 16E6(4C4S) proteins (**f**) 4C4S R84A, (**g**) 4C4S R84E, (**h**) 4C4S F76A, (**i**) 4C4S R62A. Expected molecular weights are calculated with the removal of the translational N-terminal methionine.

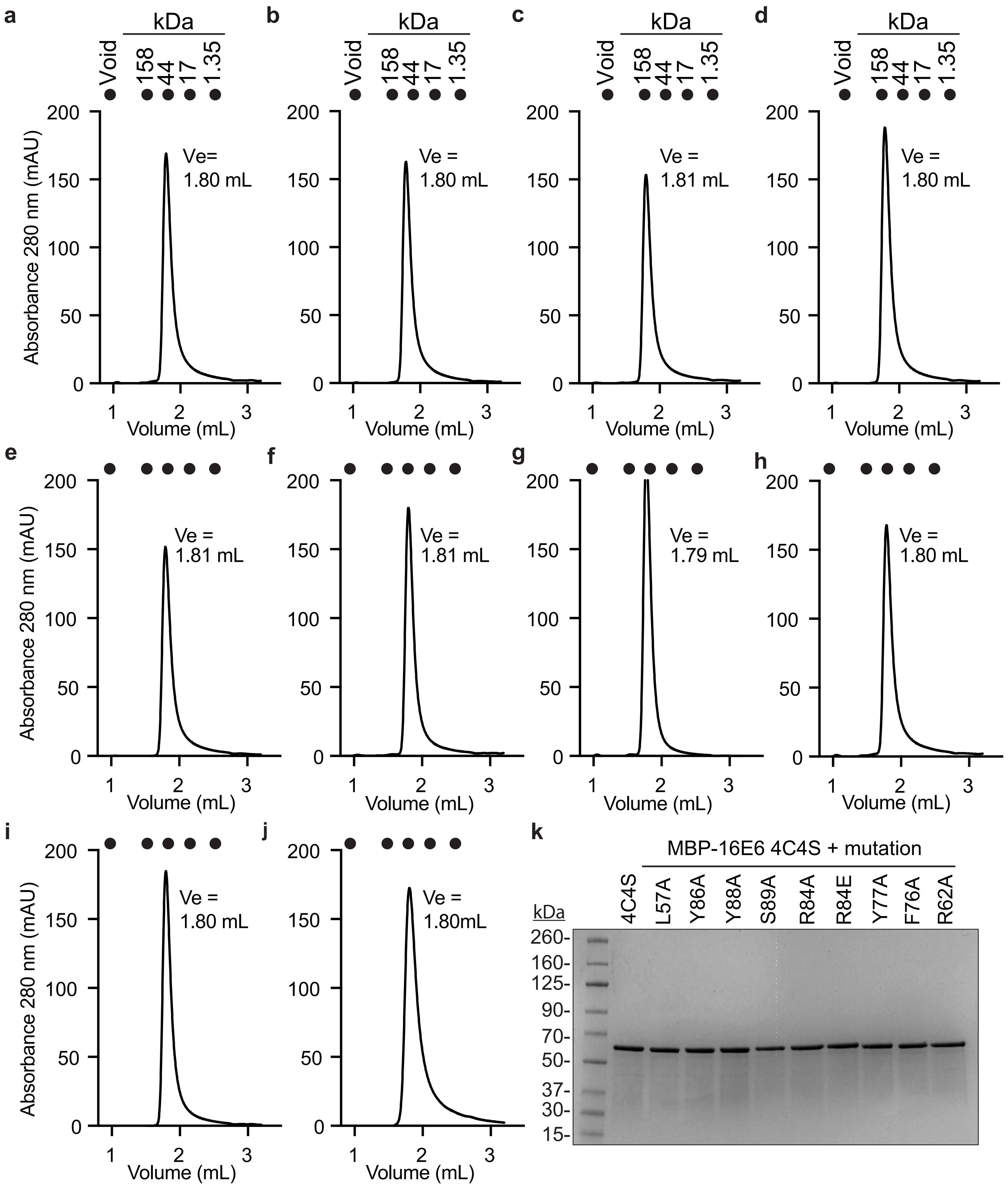

Supplementary Figure 18. Size exclusion chromatography of recombinant MBP-16E6(4C4S) proteins. (**a**) 4C4S, (**b**) 4C4S L57A, (**c**) 4C4S Y86A, (**d**) 4C4S Y88A, (**e**) 4C4S S89A, (**f**) 4C4S R84A, (**g**) 4C4S R84E, (**h**) 4C4S F76A, (**i**) 4C4S R62A and (**j**) Y77A. Elution volume, Ve, of molecular weight standards are depicted above graphs. (**k**) The indicated proteins (1 µg) were resolved by SDS-PAGE and visualized with Instant Blue stain.

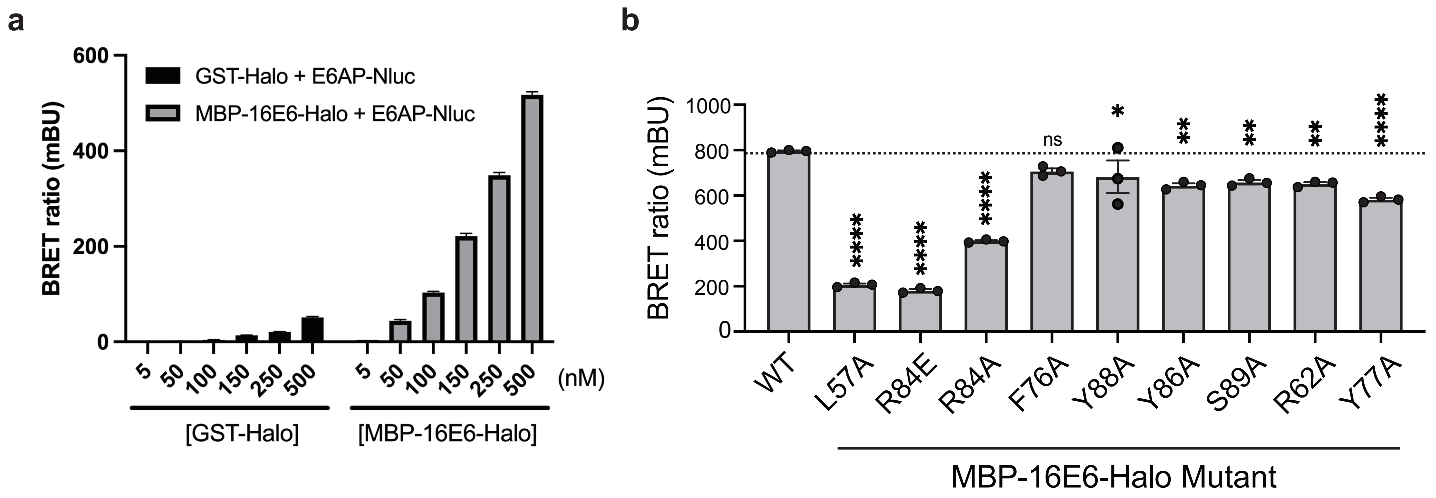

Supplementary Figure 19. Investigation of specific binding events using the in-solution NanoBRET proximity assay. (**a**) Binding specificity analysis of E6AP-Nluc (5 nM) titrated against 618-labeled MBP-16E6-Halo or GST-Halo. The assay differentiates the specific BRET pair interaction (MBP-16E6-Halo and E6AP-Nluc) from a non-specific interaction serving as a negative control (GST-Halo and E6AP-Nluc). (**b**) In-solution NanoBRET proximity assay evaluates binding events between E6AP-Nluc and 618-labeled MBP-16E6-Halo WT, as well as its mutants. Data points denote the mean ± SEM, aggregated from three independent experiments (n=3). Statistical significance is represented by p-values: L57A, R84E, R84A: **** $p < 0.0001$; F76A: $p = 0.0974$; Y88A: *$p=0.0211$; Y86A: **$p=0.0017$; S89A: **$p=0.0044$; R62A: **$p=0.0028$; Y77A: ****$p < 0.0001$; ns = not significant. The graph is averaged from three independent experiments. Significance was calculated using One-way ANOVA with Dunnett's multiple comparisons test.

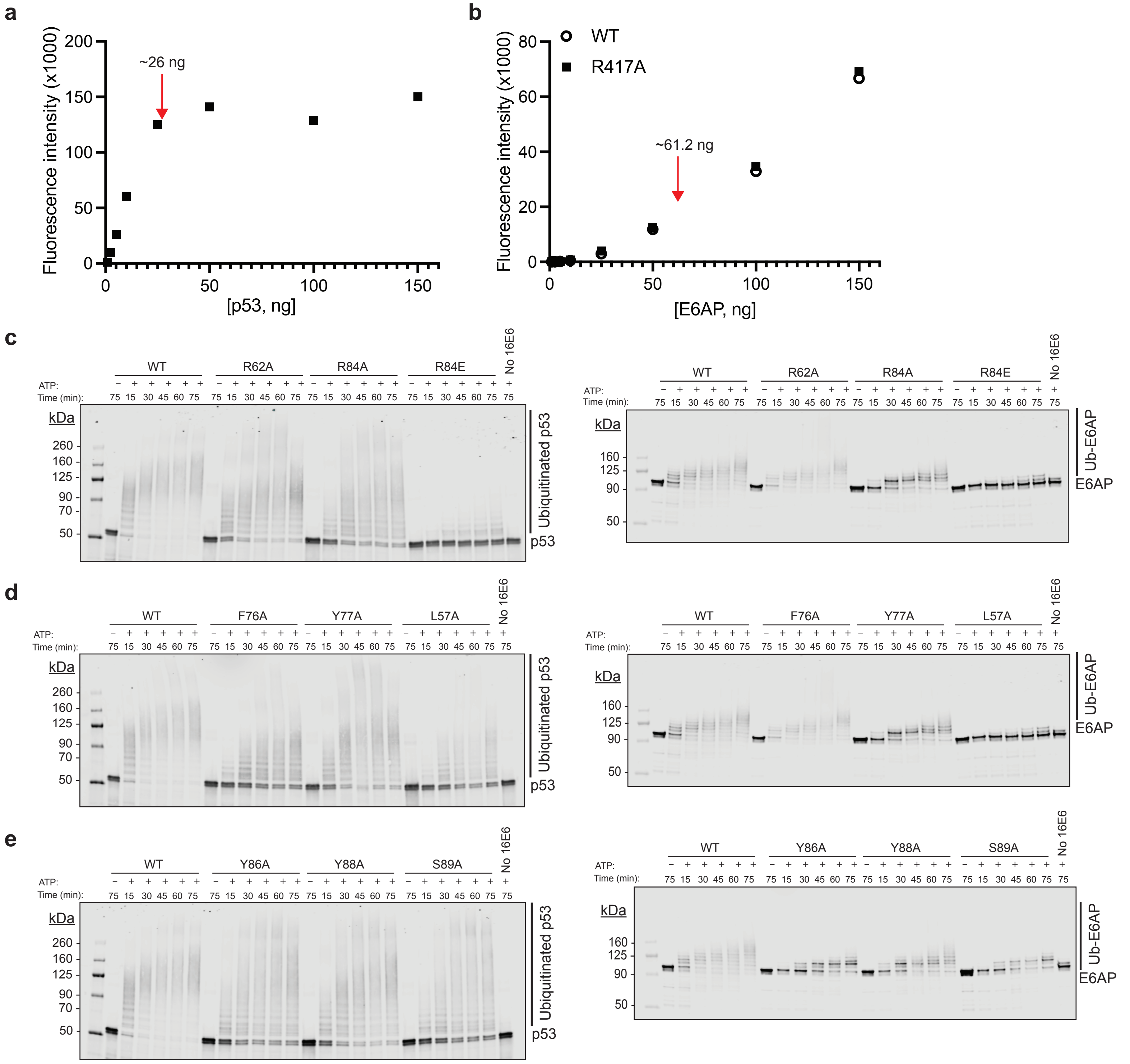

Supplementary Figure 20. Quantitative Western Blot Analysis of Recombinant p53 and E6AP Proteins. SDS-PAGE followed by western blot analysis was performed to resolve increasing concentrations of recombinant (**a**) full-length p53 and (**b**) full-length E6AP wild-type (circles) or E6AP R117 (square). Western blots were quantified to assess assay linearity. For the p53 ubiquitination assays presented in Figure 5, 26.4 ng of full-length p53 and 61.2 ng of E6AP were separated from the reaction mixture using SDS-PAGE. (**c-e**) The full Western blots from Figure 5, displaying bands for p53 (left) and E6AP (right). Left and right panels are from the same blot. Technical replicates are represented by wild-type (WT) samples. Images are representative of three independent experiments. Experimental details are described in Materials and Methods.

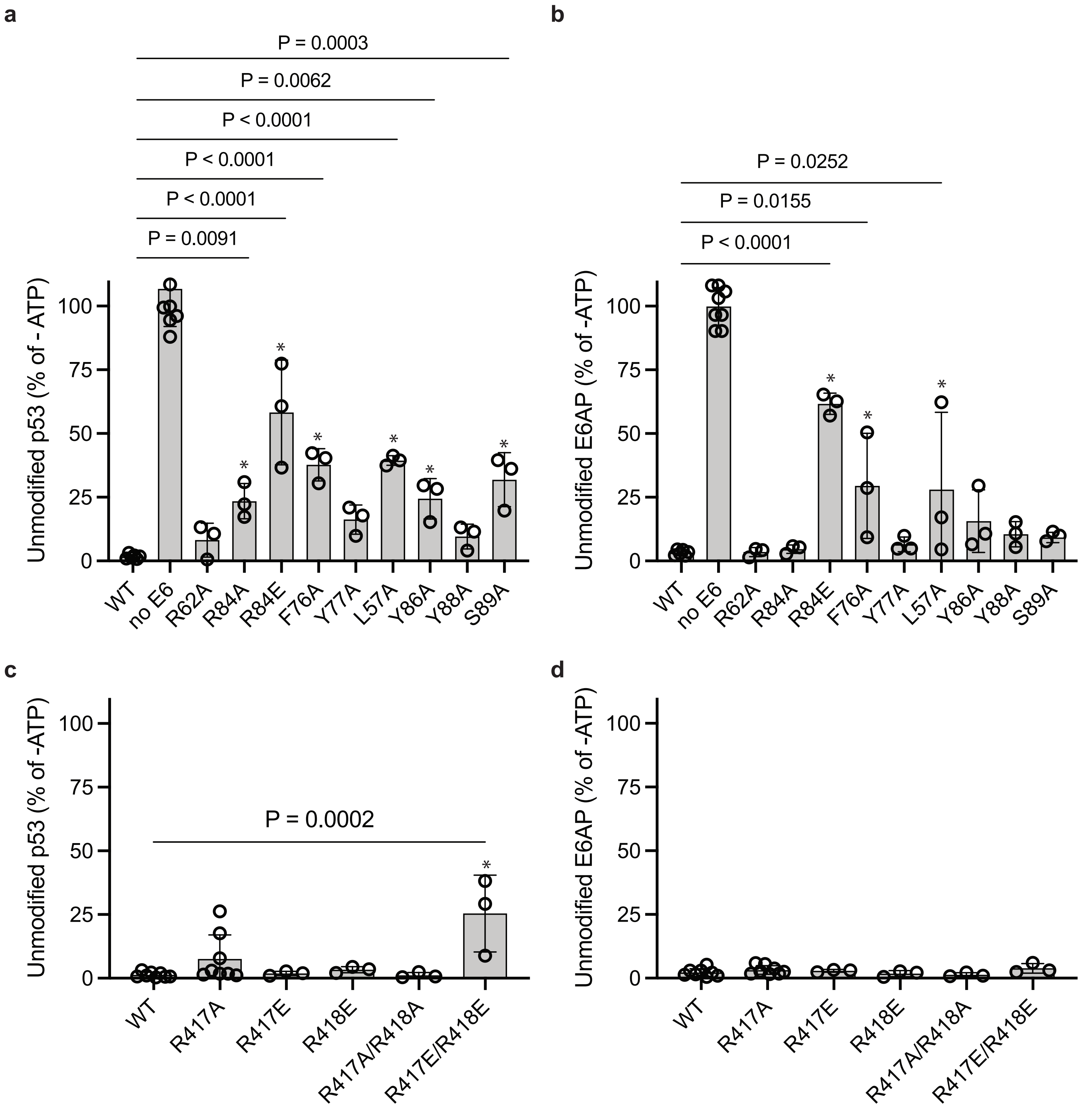

Supplementary Figure 21. Statistical analysis of the impact of MBP-16E6 or E6AP mutants on ubiquitination of p53 or E6AP at 75 min. Employing one-way ANOVA with multiple comparisons analysis, this figure assesses unmodified p53 (**a**) and E6AP (**b**) levels for both wild-type and mutant MBP-16E6(4C4S).The corresponding western blots are displayed in Figure 5 and Supplementary Figure 20. The same statistical analysis method was used to evaluate unmodified p53 (**c**) and E6AP (**d**) levels for WT and mutant E6AP. The corresponding western blots are displayed in Figure 6 & Supplementary Figure 26. The data are expressed as the mean ± standard deviation. An asterisk indicates significant values, symbolizing p-values of less than 0.05, with exact p-values indicated when applicable. Data were obtained from three independent experiments for all mutant MBP-16E6 (4C4S) proteins and six independent experiments for WT MBP-16E6 in (a) and (b). For (c) and (d) data were obtained from three independent experiments for R417E, R418E, R417A/R418A, R417E/R418E E6AP, and eight independent experiments for WT and R417A E6AP.

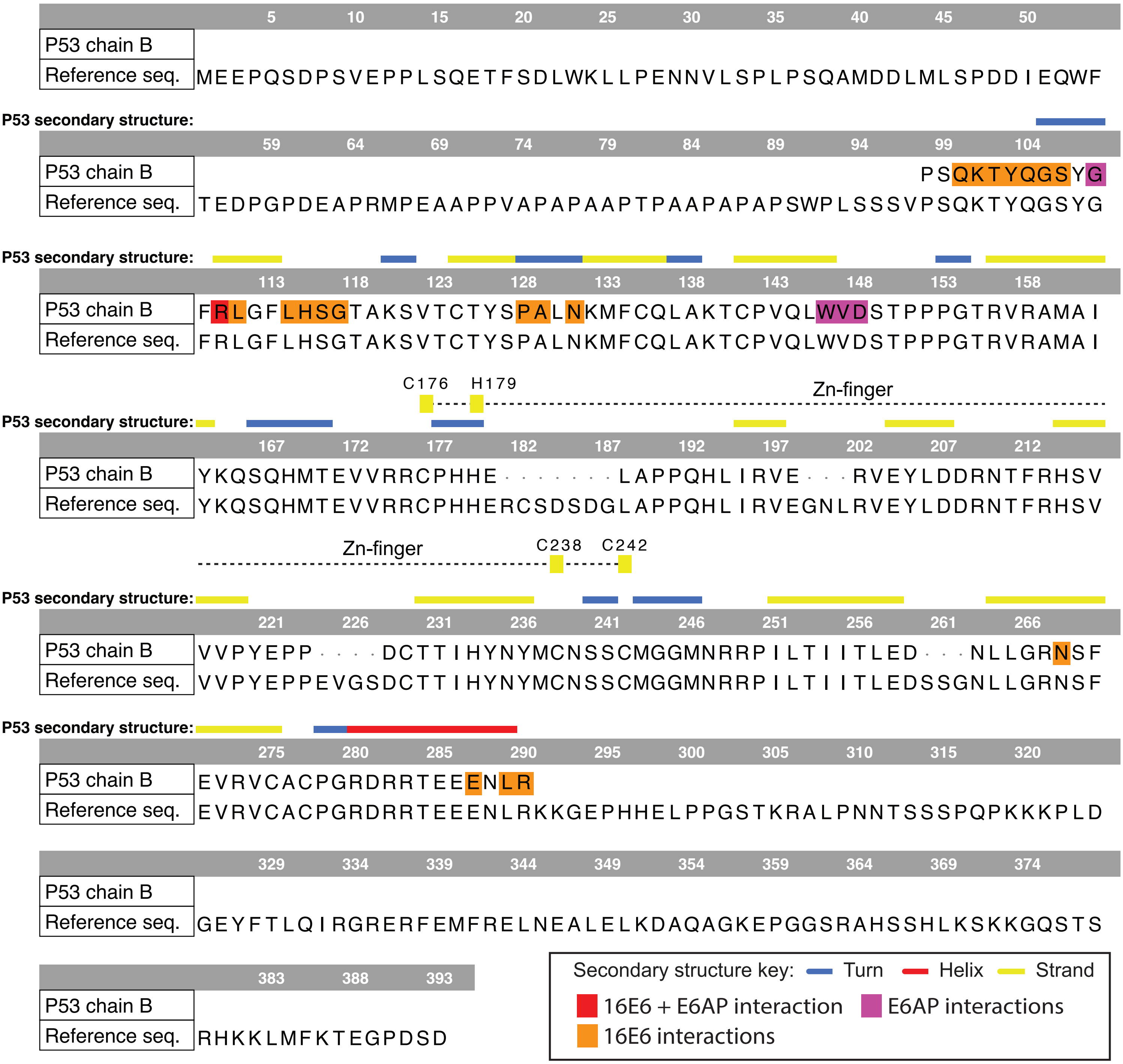

Supplementary Figure 22. Sequence alignment of canonical $p53^{core}$ sequence and resolved elements in the 16E6, $p53^{core}$, and E6AP ternary complex. The p53 reference sequence (Uniprot ID no. P04637) was aligned to the $p53^{core}$ chain B cryoEM structure. Secondary structures guided by the p53 cryoEM structure are indicated above the sequence (blue for turn, red for helix, yellow for strand). $p53^{core}$ residues that interact with 16E6 (orange), E6AP (magenta), or both (red) are highlighted.

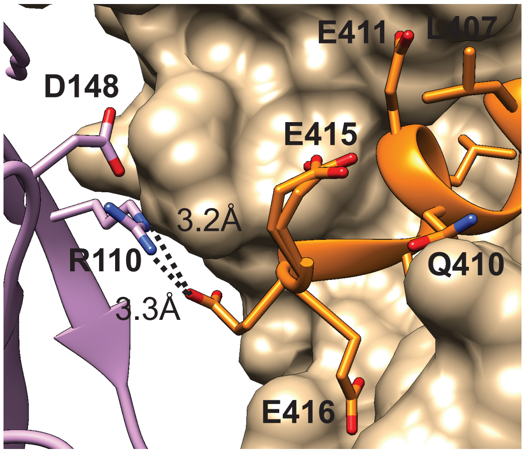

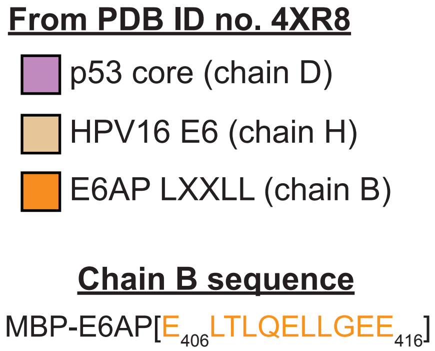

Supplementary Figure 23. Molecular interaction between the p53^{core} domain and E6AP. The interactions between the C-terminus of E6AP LXXLL peptide (orange, stick representation) from PDB ID no. 4XR8, highlighting the hydrogen bonding interactions with p53 R110 (purple, stick representation) and E6AP E416 on the LXXLL motif (orange, ribbon representation).

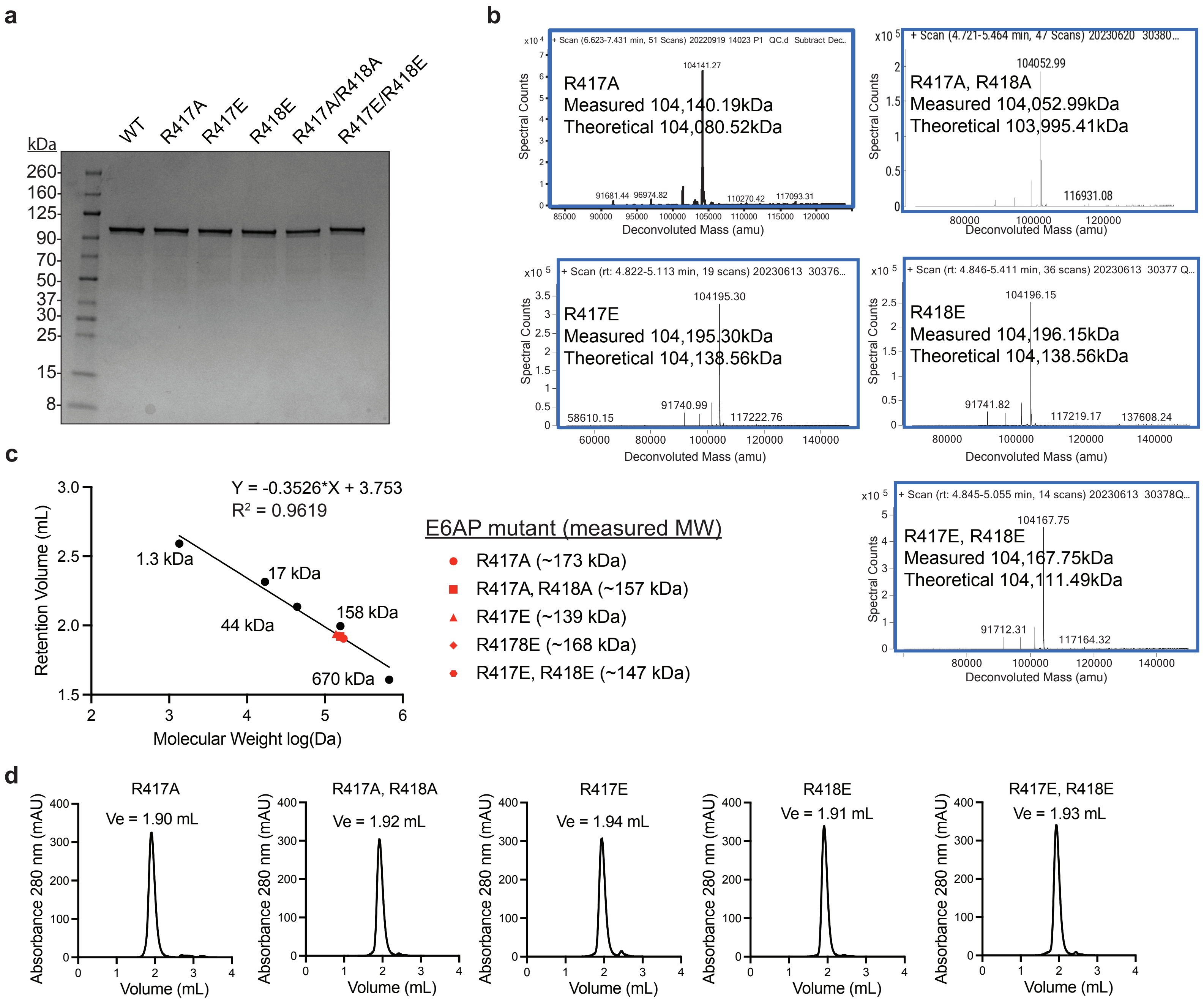

Supplementary Figure 24. Quality control of E6AP mutants predicted to interfere with p53 binding. (**a**) SDS-PAGE analysis of the specified E6AP proteins (1 µg each), with protein bands visualized using Instant Blue stain. (**b**) LC-MS deconvoluted intact mass spectra (**c**) A linear regression calibration curve (black line) derived from BioRad Gel Filtration Standard (BioRad, cat. no. 1511901; represented by black circles) using a Superdex200 5/150 size exclusion column, mapping retention volume to the molecular weight of the standards. (**d**) Size exclusion chromatography profiles of E6AP mutants, with their peak retention volumes (Ve) plotted on the calibration curve from (c) as red shapes, indicating their molecular weight.

a

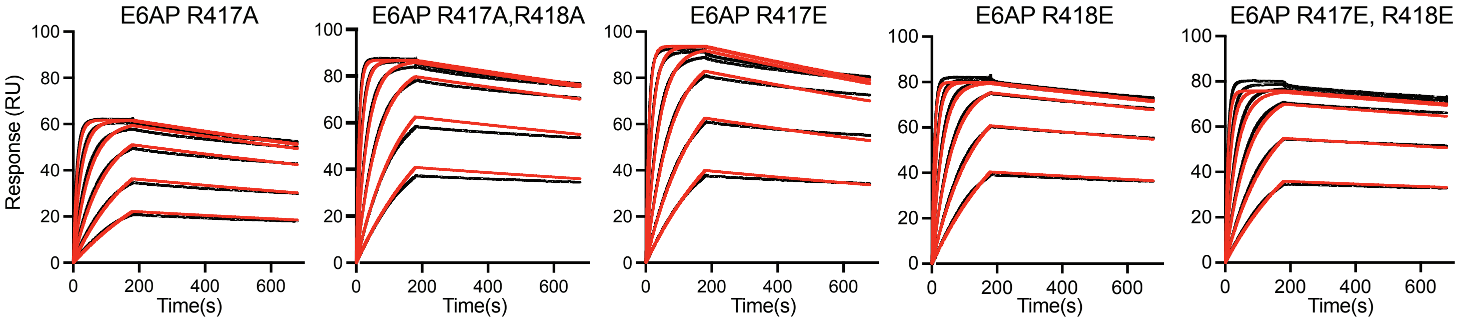

b

E6AP Mutants Kinetics Table

Capture Ligand	Analyte Solution	Replicate	K_a ($M^{-1}s^{-1}$)	SE (K_a) ±	K_d (s^{-1})	SE (K_d) ±	mean K_D (nM)	SE (K_D) ±
E6AP R417A	16E6(4C4S)	1	1.69E+06	6.60E+02	3.79E-04	4.20E-07	0.216	0.008
		2	1.64E+06	7.00E+02	3.67E-04	4.50E-07		
		3	1.66E+06	7.00E+02	3.30E-04	4.40E-07		
E6AP R417A ,R418A	16E6(4C4S)	1	2.34E+06	1.00E+03	2.67E-04	4.20E-07	0.116	0.005
		2	2.40E+06	3.40E+03	2.59E-04	3.60E-07		
		3	2.41E+06	4.50E+03	3.00E-04	4.90E-07		
E6AP R417E	16E6(4C4S)	1	2.01E+06	7.00E+02	3.06E-04	3.50E-07	0.162	0.005
		2	2.04E+06	9.00E+02	3.38E-04	4.50E-07		
		3	2.05E+06	9.80E+02	3.44E-04	4.80E-07		
E6AP R418E	16E6(4C4S)	1	2.93E+06	5.70E+03	2.11E-04	4.40E-07	0.092	0.018
		2	2.78E+06	4.00E+03	2.14E-04	3.30E-07		
		3	2.26E+06	1.00E+03	2.91E-04	4.50E-07		
E6AP R417E,418E	16E6(4C4S)	1	2.63E+06	4.30E+03	1.18E-04	3.60E-07	0.068	0.013
		2	2.37E+06	4.40E+03	1.60E-04	4.40E-07		
		3	2.13E+06	3.30E+03	1.92E-04	3.80E-07		

Supplementary Figure 25. The evaluation of rationally designed E6AP mutations predicted to interfere with p53 recruitmentby surface plasmon resonance (SPR) using S CAP chips. (**a**) SPR sensograms of recombinant MBP-16E6(4C4S) protein binding to biotinylated E6AP mutants starting from 50 nM in 2-fold serial dilution. Measured binding responses (black) with curve fits to a 1:1 binding model (red) are displayed. Plots are representative of three experimental replicates. (**b**) Summary of three experimental replicates fitted to a 1:1 binding model. Abbreviations used include RU for response units, K_D for the dissociation constant, K_a for the association rate, K_d for the dissociation rate, and SE for the standard error of the mean.

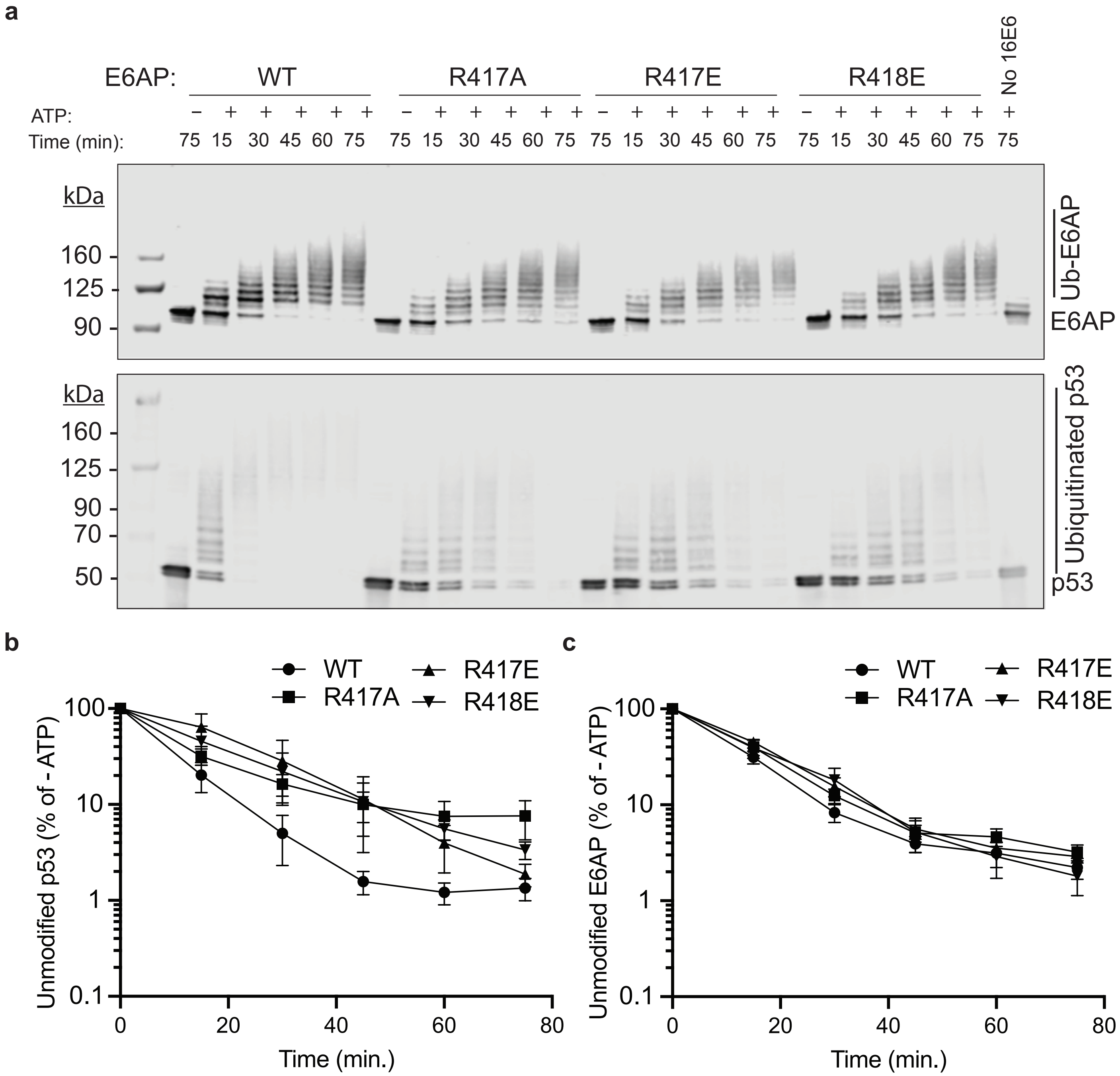

Supplementary Figure 26. Single E6AP point mutations at the p53 interaction site minimally affect p53 ubiquitination while E6AP auto-ubiquitination is unchanged. (**a**) Ubiquitination assays were conducted by incubating MPB-16E6(4C4S) with wild-type or mutant E6AP and full-length p53, in the presence or absence of ATP. Results were resolved by SDS-PAGE and visualized by western blot for E6AP (upper) and p53 (lower). Quantification of unmodified (**b**) full-length p53 and (**c**) E6AP are presented. All values represent the mean ± SEM from three independent experiments for R417E and R418E, and eight independent experiments for WT and R417A E6AP. For (b) and (c) WT E6AP is represented by a closed circle, R417A by a closed square, R417E by a closed triangle, and R418E by a closed upside down triangle.

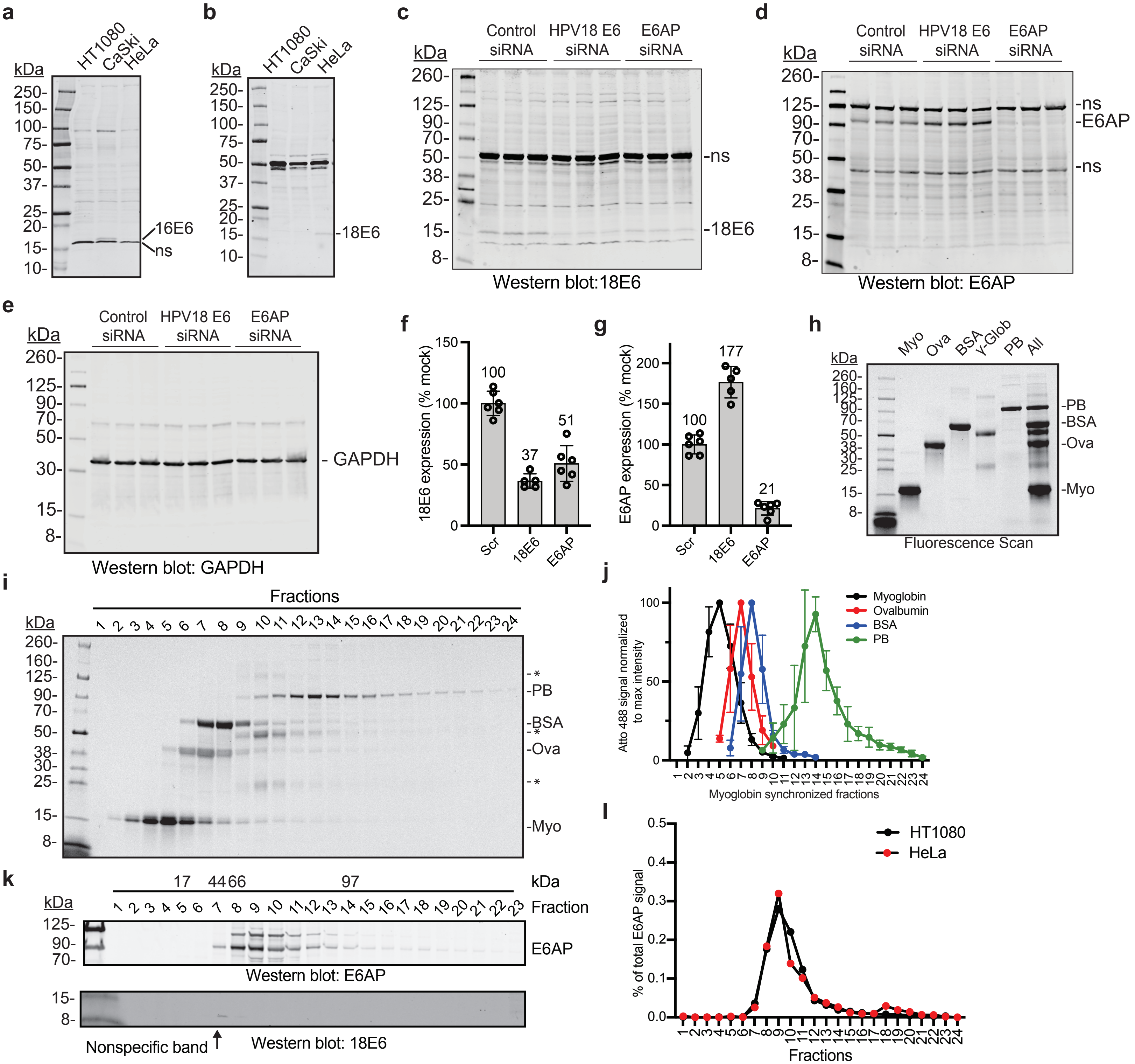

Supplementary Figure 27. HPV E6 Antibody Validation, Sedimentation Gradient Standards, and Sedimentation Gradient Runs. (**a**) HT1080 (HPV-), CaSki (HPV16+), or HeLa (HPV18+) cell lysates were resolved by SDS-PAGE and blotted for 16E6, and (**b**) for 18E6. NS, non specific. Sedimentation gradient was not performed for HPV16 cells as the antibody recognizes a non-specific band near the molecular weight of 16E6. HeLa cells treated with control siRNA or siRNA targeting 18E6 or E6AP and lysates were resolved by SDS-PAGE then blotted for (**c**) 18E6, (**d**) E6AP or (**e**) GAPDH. Blots from (c and d) were normalized to total protein loading by Ponceau and (**f**) 18E6 or (**g**) E6AP protein levels quantified. Three replicates for each siRNA are depicted in (d and e), however five replicates for 18E6 and six replicates for E6AP and control siRNA were run, analyzed, and included in (f and g). Values represent the mean ± SD. (**h**) Proteins were conjugated with Atto-488 and resolved by SDS-PAGE, then imaged at 488 nm. (**i**) Proteins from (h), were combined and subjected to sedimentation gradient, then imaged at 488 nm as described in the Materials and Methods. γ-globulin was degraded and is indicated by an "*". (**j**) Data from (i) was quantified and fractions were synchronized to myoglobin, Gamma globulin was excluded due to degradation. Values represent the mean ± SD of four independent experiments. (**k**) Lysate from the HPV-negative cell line HT1080 was separated by sedimentation gradient and blotted for E6AP (top) or 18E6 (bottom). As expected, 18E6 is not present, although a non-specific low molecular weight band is observed, which is also found in HPV+ cell lines. (**l**) Quantification of the E6AP signal from (h) superimposed with the data from Figure 7b demonstrates that the E6AP sedimentation profile is consistent between HPV+ and HPV negative cell lines. Values represent the mean of two independent experiments.

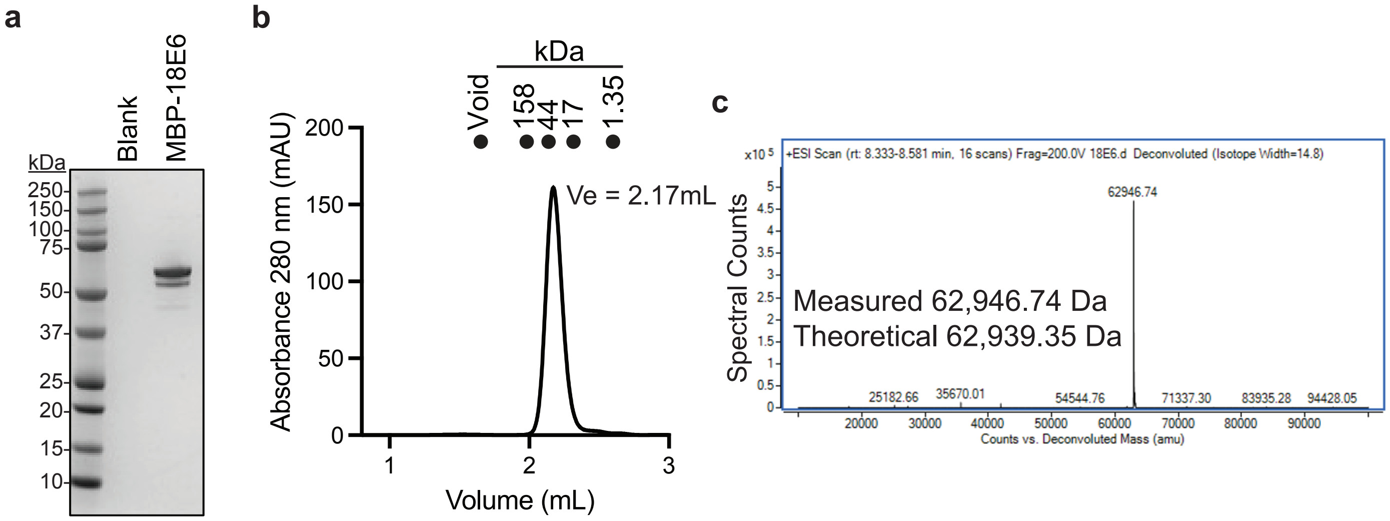

Supplementary Figure 28. Quality assessment of the MBP-18E6 protein. (**a**) SDS-PAGE analysis of the provided MBP-18E6 protein (2 µg) stained with Instant Blue. (**b**) Elution profile of MBP-18E6 on a Superdex200 5/150 size exclusion column, indicating the Retention Volume (Ve). (**c**) LC-MS deconvoluted intact mass spectra for MBP-18E6.

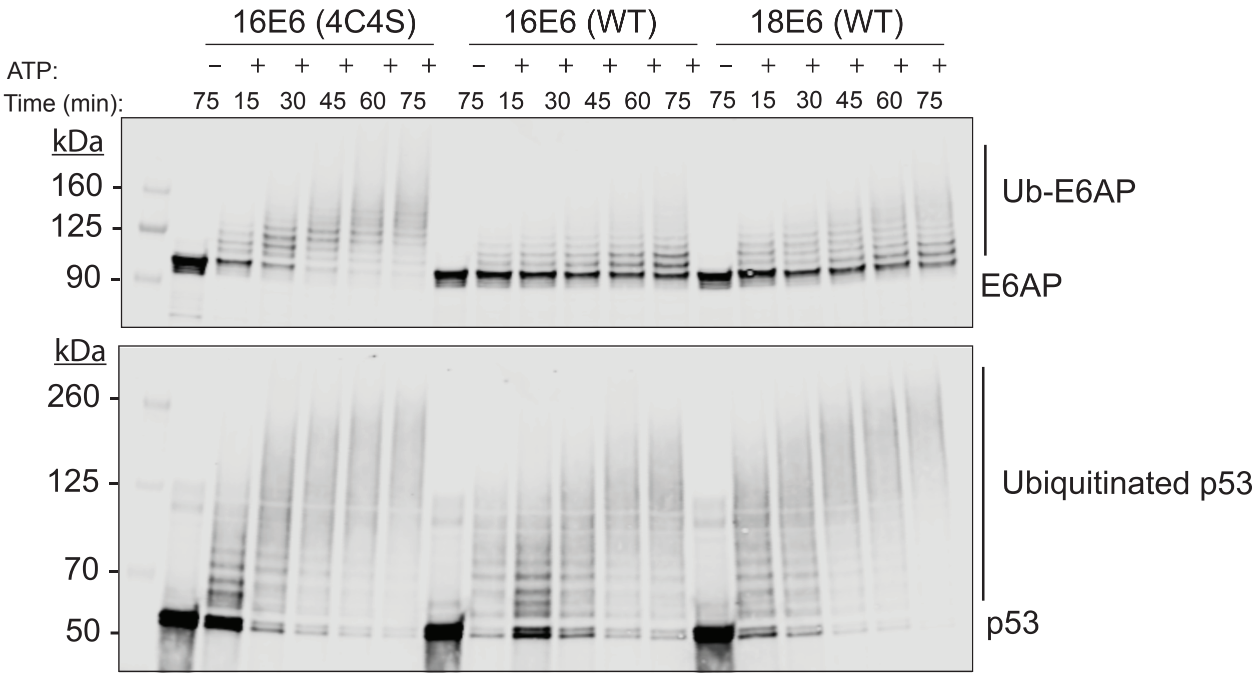

Supplementary Figure 29. MBP-16E6 (4C4S), 16E6 (WT), and MBP-18E6 (WT) stimulate full-length p53 and E6AP ubiquitination. Incubation of MBP-16E6(4C4S), MBP-16E6(WT), or MBP-18E6(WT) with WT full-length E6AP, and full-length p53 in the presence or absence of ATP, resolved by SDS-PAGE, and visualized by western blot for E6AP (upper blot) or full-length p53 (lower blot). Some deviation of MBP-16E6(WT) is attributed to protein aggregation known to occur with this protein. Assay details are described in the Materials and Methods section. Data are representative of two independent experiments.

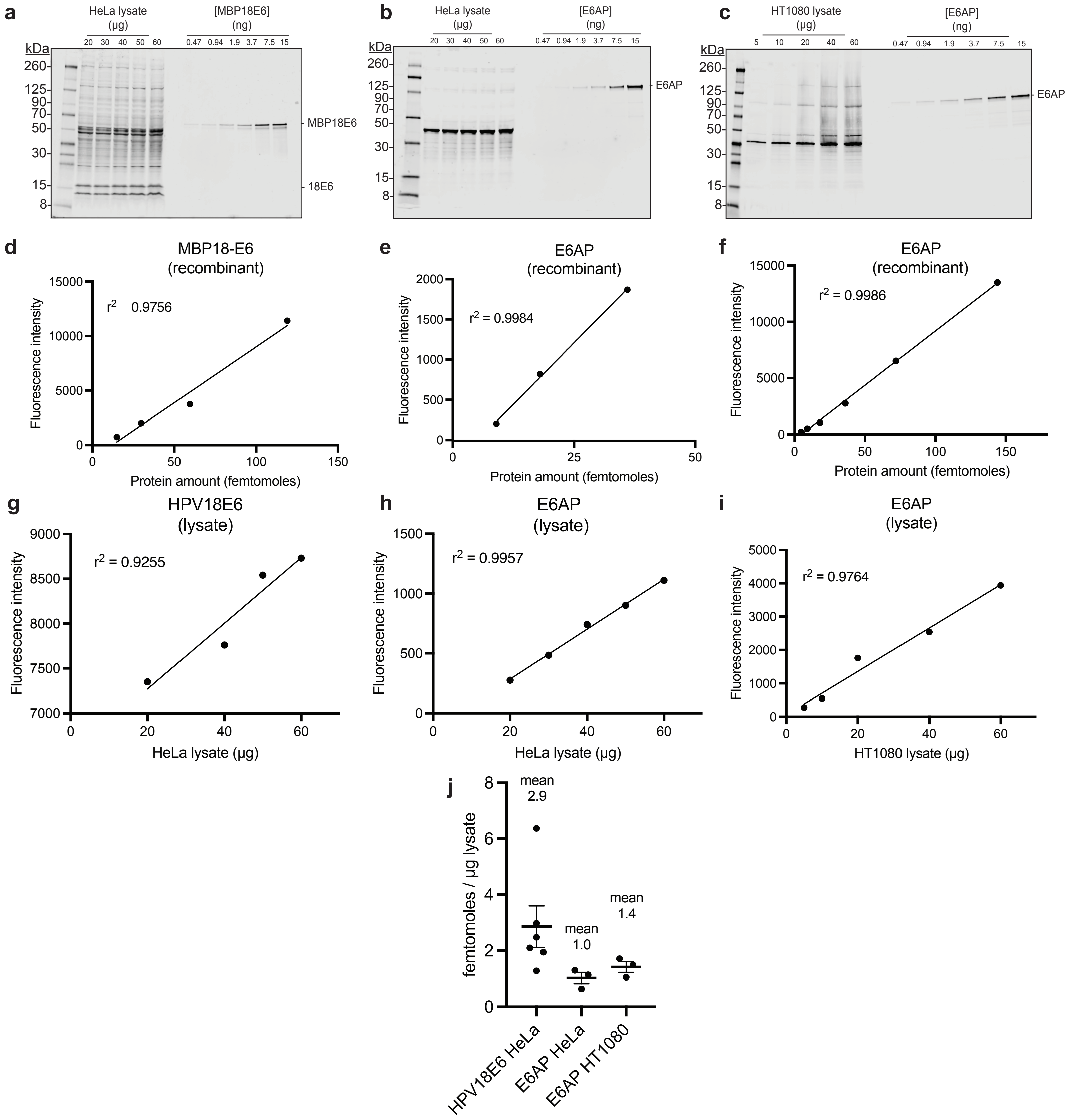

Supplementary Figure 30. Semi-quantitative analysis of the molar amount of 18E6 and E6AP proteins in HeLa and HT1080 lysates. HeLa (HPV18+) (**a** and **b**) and HT1080 (HPV-) (**c**) cell lysates and purified, recombinant MBP-18E6, or E6AP, were resolved by SDS-PAGE and blotted for HPV18 E6 or E6AP, respectively. Fluorescence intensities for each of the bands for recombinant MBP-18E6 and E6AP were plotted against the protein amount (converted to femtomoles), for HeLa (**d** and **e**) and HT1080 (**f**). The fluorescence intensities for each HPV18E6 (**g**), E6AP from HeLa (**h**), and E6AP from HT1080 (**i**) were also plotted and the protein amount (in femtomoles) was interpolated from the standard curve generated in (**d-f**). Blots shown and the data plotted are from one experiment and are representative of three independent experiments for E6AP quantification in HeLa and HT1080, and six independent experiments for HPV18 E6 in HeLa. Interpolated protein values were calculated as an amount per µg of lysate and the mean from each experiment was calculated and values plotted in (**j**) with each data point, the mean, and the standard error of the mean indicated.

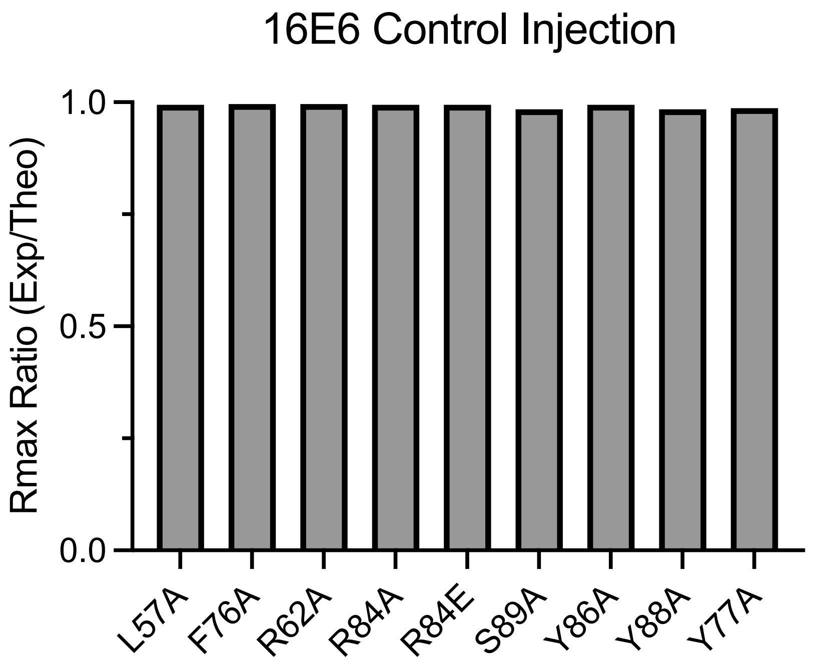

Supplementary Figure 31. Control injections of wildtype 16E6 onto the conditioned biotinylated-E6AP sensor chip surface used for each mutant 16E6 surface plasmon resonance experiment. An injection of 20 nM 16E6(4C4S) wildtype at 30 µL/min for 180 seconds over a CAP-sensor chip immobilized with biotinylated E6AP to measure the maximum binding signal, Experimental Rmax. The Theoretical Rmax is derived from the equation Theoretical Rmax = (MW analyte / Mw ligand) x Response Immoblized Ligand. The ratio of Experimental Rmax to Theoretical Rmax is plotted to demonstrate that E6AP retained full binding potential for experiments involving 16E6 mutant kinetics.

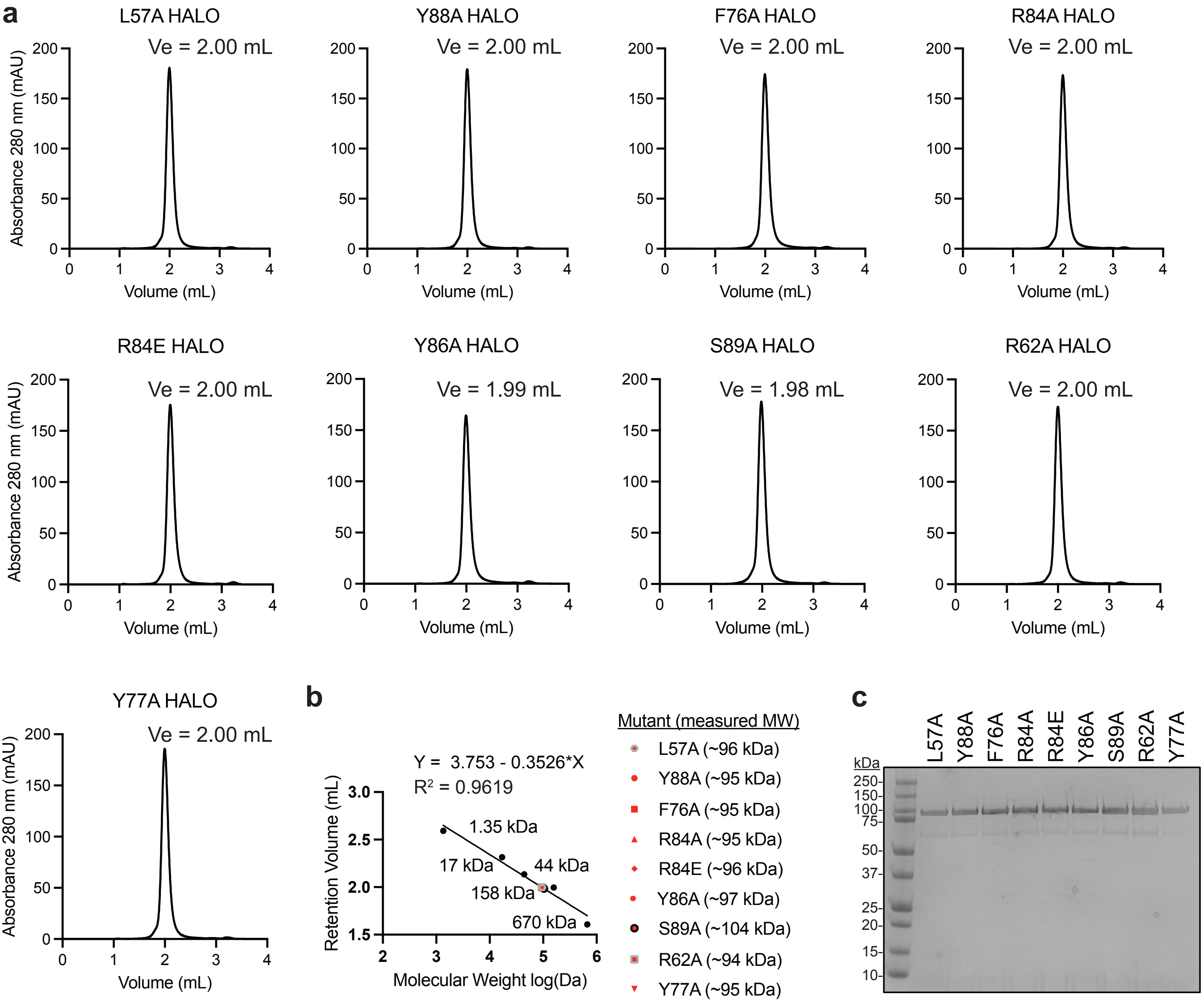

Supplementary Figure 32. Quality control of MBP-16E6-Halo mutant proteins. (**a**) Resolution of 16E6-Halo proteins over an analytical Superdex200 5/150 size exclusion column. Retention Volume, Ve. (**b**) Gel filtration standard (Biorad, Cat no. 1511901; represented by black circles) with peak retention volumes plotted against each standard's respective molecular weight on the same column. Linear regression (black line) was used to prepare the calibration curve and retention volumes of MBP-16E6-Halo mutant proteins were fitted (represented by red/gray shapes) to approximate molecular weight. (**c**) The indicated MBP-16E6-Halo mutants (1 µg) were resolved by SDS-PAGE and visualized with Instant Blue stain.

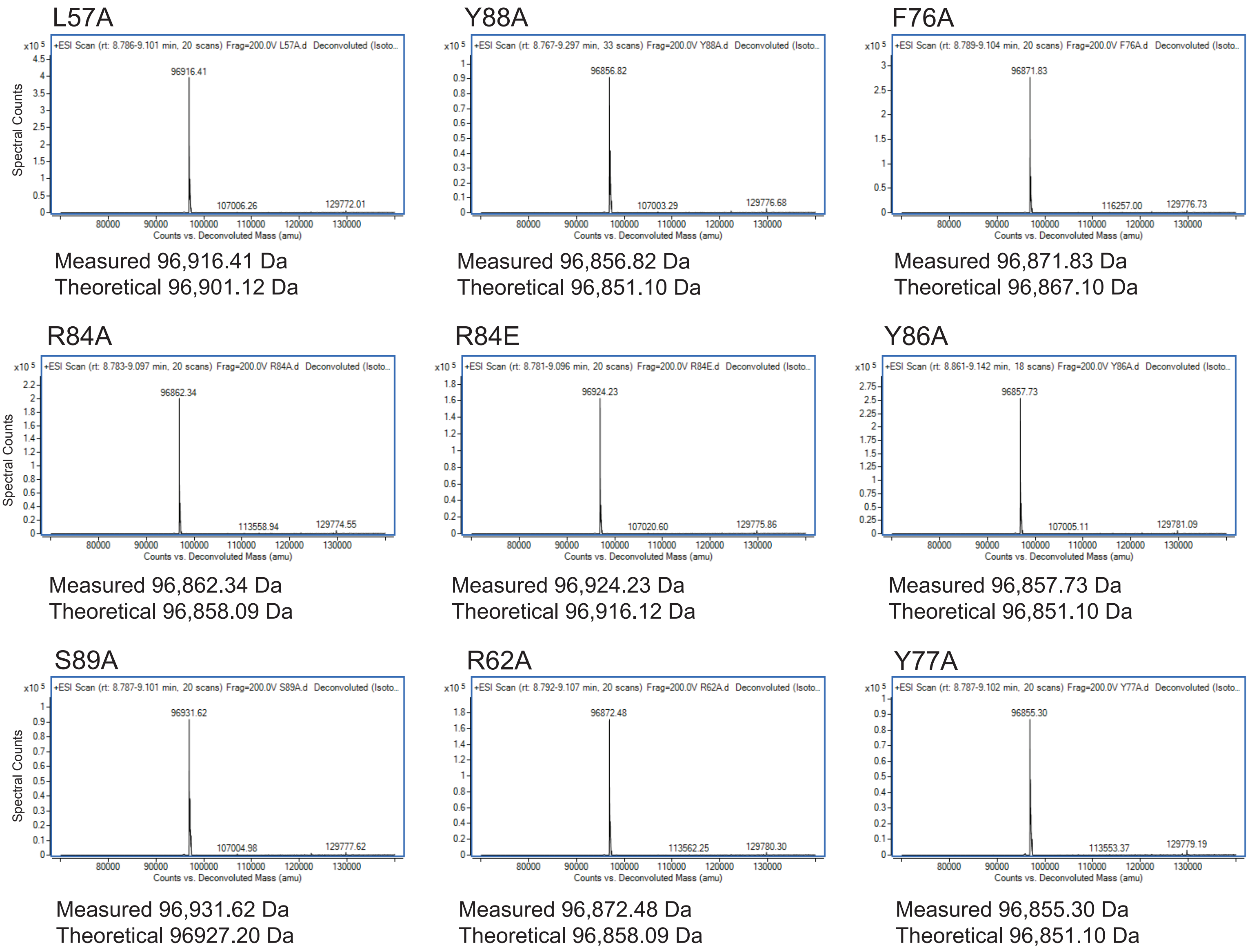

Supplementary Figure 33. Characterization of MBP-16E6(4C4S)-Halo nanoBRET proteins using LC-MS deconvoluted intact mass spectra.

Supplementary Table 1. Cryo-EM data collection, refinement, and validation statistics

**E6AP/E6/p53 complex**	PDB ID: 8GCR<br>EMDB ID: EMD-29941
**Data collection and Processing**	
Microscope	Titan Krios
Voltage (kV)	300
Camera	Gatan K3 Summit
Magnification	105,000
Pixel size at detector (Å/pixel)	0.83
Total electron exposure ($e^{-}/Å^2$)	80.5
Number of frames collected during exposure	50
Defocus range (µm)	-1.5 ~ -2.5
Automation software	SerialEM
Tilt angle	0
Energy filter slit width (eV)	20
Micrographs collected (no.)	8,127
Symmetry imposed	C1
Initial particle images (no.)	2,349,301
Final particle images (no.)	105,794
FSC threshold	0.143
Map resolution (Å)	3.38
Map resolution range (Å)	3.33 ~ 4.24
**Refinement**	
Initial model used (PDB code)	4XR8
Model Resolution (Å)	3.62/3.38
FSC threshold	0.5/0.143
Map sharpening *B* factor ($Å^2$)	-113
Model Composition	
Non-hydrogen atoms	7206
Protein residues	877
Ligands	3
B factors ($Å^2$)	
Protein	19.75
Ligand	30.89
R.m.s deviations	
Bond lengths (Å)	0.006
Bond angles (°)	1.073
Validation	
MolProbity score	1.76
Clashscore	8.31
Poor rotamers (%)	0.00
Ramachandran plot	
Favored (%)	95.57
Allowed (%)	4.43
Disallowed (%)	0.00

Supplementary Table 2. MD simulations of E6AP in different states.

#	System	Template	Size (atoms #, box)	Duration
1	16E6-E6AP-P53	cryoEM	# Water =~61K<br># Na = 197<br># Cl = 182<br>Box =126.2* 126.2 * 126.2 $Å^3$	~1000 ns
2	16E6-E6AP (LXXLL peptide)-P53	cryoEM	# Water =~23K<br># Na = 69<br># Cl = 79<br>Box =91.2* 91.2 * 91.2 $Å^3$	~250 ns
3	16E6-E6AP	cryoEM	# Water =~61K<br># Na = 200<br># Cl = 179<br>Box =125.7* 125.7 * 125.7 $Å^3$	~200 ns
4	apo-E6AP	cryoEM	# Water =~56K<br># Na = 192<br># Cl = 164<br>Box =126.2* 126.2 * 126.2 $Å^3$	~200 ns
5	apo-E6AP	AF2	# Water =~61K<br># Na = 197<br># Cl = 182<br>Box =122.0* 122.0 * 122.0 $Å^3$	~200 ns

Supplementary Table 3. Molecular Dynamics Reporting Document

**Reliability and reproducibility checklist for molecular dynamics simulations**<br>***All boxes must be marked YES by acceptance unless an N/A option is available**	**Yes**	**N/A**	**Response**<br>**(Please state where this information can be found in the text)**
**1. Convergence of simulations and analysis**			
1a. Is an evaluation presented in the text to show that the property being measured has equilibrated in the simulations (*e.g.* time-course analysis)?	☒		
1b. Then, is it described in the text how simulations are split into equilibration and production runs and how much data were analyzed from production runs?	☒		
1c. Are there at least 3 simulations per simulation condition with statistical analysis?	☒		
1d. Is evidence provided in the text that the simulation results presented are independent of initial configuration?	☒		
**2. Connection to experiments**			
2a. Are calculations provided that can connect to experiments (*e.g.* loss or gain in function from mutagenesis, binding assays, NMR chemical shifts, J-couplings, SAXS curves, interaction distances or FRET distances, structure factors, diffusion coefficients, bulk modulus and other mechanical properties, *etc*.)?	☒		
**3. Method choice**			
3a. Is it described in the text what force field and water model are used and why?	☒		
3b. Do simulations contain membranes, membrane proteins, intrinsically disordered proteins, glycans, nucleic acids, polymers, or cryptic ligand binding?	☒	☐	Response not needed if **N/A**
If 3b is **YES**, are enhanced sampling methods used?	☐	☐	N/A. E6 is an intrinsic disordered protein but would be stabilized after forming complex with E6AP and p53
If enhanced sampling methods are used, are the convergence criteria clearly stated?	☐		
If 3b is **YES**, is it explained in the text why or why not enhanced sampling methods are used?	☐		N/A. As MD-simulation was used to demonstrate the flexibility of E6 LXXLL motif without E6AP binding.
**4. Code and reproducibility**			
4a. Is a table provided describing the system setup, such as simulation box dimensions, total number of atoms, total	☒		

number of water molecules, salt concentration, lipid composition (number of molecules and type)?			
4b. Is it described in the text what simulation and analysis software and which versions are used?	☒		
4c. Are initial coordinate and simulation input files and a coordinate file of the final output provided as supplementary files or in a public repository?	☒		
4d. Is there custom code or custom force field parameters?	☐	☒	Response not needed if **N/A**
If **YES**, are they provided as supplementary profiles or in a public repository?	☐		
