# Supplementary material for: Structure of the p53 degradation complex from HPV16
Source: Nat Commun. 2024 Feb 28;15:1842. doi: 10.1038/s41467-024-45920-w (PMC10902388; doi:10.1038/s41467-024-45920-w)
Supplement: Supplementary file 3 — Description of Additional Supplementary Files [file 41467_2024_45920_MOESM3_ESM.pdf]

Description of Additional Supplementary Files

File name: Supplementary Data 1
Description: Individual protein contacts from the protein contact utility in MOE was used to define interactions using the default settings and a summary of all interactions in Excel file format.

File name: Supplementary Data 2
Description: The protonated 16E6, E6AP, and $p53^{core}$ ternary complex was exported in the MOE file format.

File name: Supplementary Data 3
Description: The protonated 16E6, E6AP, and $p53^{core}$ ternary complex was exported in the PDB file format.
