# Supplementary material for: Structure of the p53 degradation complex from HPV16
Source: Nat Commun. 2024 Feb 28;15:1842. doi: 10.1038/s41467-024-45920-w (PMC10902388; doi:10.1038/s41467-024-45920-w)
Supplement: Supplementary file 7 — Reporting Summary [file 41467_2024_45920_MOESM7_ESM.pdf]

nature portfolio

Corresponding author(s): Aaron H. Nile, PhD
Qi Hao, PhD

Last updated by author(s): Jan 5, 2024

Reporting Summary

Nature Portfolio wishes to improve the reproducibility of the work that we publish. This form provides structure for consistency and transparency in reporting. For further information on Nature Portfolio policies, see our Editorial Policies and the Editorial Policy Checklist.

Statistics

For all statistical analyses, confirm that the following items are present in the figure legend, table legend, main text, or Methods section.

n/a	Confirmed	
☐	☒	The exact sample size (*n*) for each experimental group/condition, given as a discrete number and unit of measurement
☐	☒	A statement on whether measurements were taken from distinct samples or whether the same sample was measured repeatedly
☐	☒	The statistical test(s) used AND whether they are one- or two-sided<br>*Only common tests should be described solely by name; describe more complex techniques in the Methods section.*
☐	☒	A description of all covariates tested
☒	☐	A description of any assumptions or corrections, such as tests of normality and adjustment for multiple comparisons
☐	☒	A full description of the statistical parameters including central tendency (e.g. means) or other basic estimates (e.g. regression coefficient) AND variation (e.g. standard deviation) or associated estimates of uncertainty (e.g. confidence intervals)
☐	☒	For null hypothesis testing, the test statistic (e.g. *F*, *t*, *r*) with confidence intervals, effect sizes, degrees of freedom and *P* value noted<br>*Give P values as exact values whenever suitable.*
☒	☐	For Bayesian analysis, information on the choice of priors and Markov chain Monte Carlo settings
☒	☐	For hierarchical and complex designs, identification of the appropriate level for tests and full reporting of outcomes
☒	☐	Estimates of effect sizes (e.g. Cohen's *d*, Pearson's *r*), indicating how they were calculated

Our web collection on statistics for biologists contains articles on many of the points above.

Software and code

Policy information about availability of computer code

Data collection

All software used to collect data is commercially available. Molecular dynamics simulations were conducted with GROMACS-2021, employing the Charmm36m parameter set for proteins and ions, and the CHARMM TIP3P model for water. Western blot images were captured with an ODYSSEY CLx LI-COR instrument using Image Studio (version 5.2).For mass photometry experiments Refeyn AcquireMP 2.3 was used to acquire data and histograms were generated, and data analyzed, using Refeyn DiscoverMP 2.3. 16E6 SPR data collection was carried out using a Biacore T200 (Cytiva, cat no. 28975001) using Biacore T200 Control Software Version 3.2.1 while 18E6 SPR data was collected using a Biacore 8K+ (Cytiva, cat no. 29344964) using Biacore Insight Control Software Version 5.0.18. MBP-tagged HPV16 E6 mutants were analyzed on a Superdex200 Increase 5/150 GL column (Cytiva, cat no. 28990945) using an Agilent 1200 Series Infinity II HPLC equilibrated in PBS, 1 mM DTT at room temperature using OpenLab CDS version C.01.10. All purification steps were done on FPLC (GE Healthcare, AKTA FPLC) using UNICORN version 7.10 control software. Nano-Glo Luciferase Assay Substrate (Promega N113A) was added to the mixture with a final dilution of 1:200. Protein mixture (20 µl) was transferred to an Optiplate 384-well, white Opaque 384-well microplate (Perkin Elmer 6007290) and the donor emission (460 nm) and acceptor emission (618 nm) were measured immediately on the ClarioStar plate reader (BMG Labtech) using the MARS software (version 3.42 R4).

Data analysis	All analyses were performed using non-proprietary software, detailed in the Materials and Methods section. The manuscript was composed in Microsoft Office (version 16.56), and figures designed in Adobe Illustrator (version 26.1). Protein structures were visualized using UCSF Chimera (version 1.14), UCSF Chimera X (version 1.4), PyMOL (version 2.5.2), and MOE (version 2020.09.01). Western blot images were quantified with an ODYSSEY CLx LI-COR instrument using Image Studio (version 5.2). Surface Plasmon Resonance data were analyzed using Biacore T200 Evaluation Software (version 3.2.1).Movie alignment and dose-weighting were executed with MotionCor2 via RELION (version 3.0.8), and processed images were further analyzed using cryoSPARC (v3.0.1). Binding affinity measurements via mass photometry were assessed with the DiscoverMP v2023 R2 software.

For manuscripts utilizing custom algorithms or software that are central to the research but not yet described in published literature, software must be made available to editors and reviewers. We strongly encourage code deposition in a community repository (e.g. GitHub). See the Nature Portfolio guidelines for submitting code & software for further information.

Data

Policy information about availability of data

All manuscripts must include a data availability statement. This statement should provide the following information, where applicable:

- Accession codes, unique identifiers, or web links for publicly available datasets
- A description of any restrictions on data availability
- For clinical datasets or third party data, please ensure that the statement adheres to our policy

Cryo-EM data for the 16E6/E6AP/p53 complex is available in the Electron Microscopy Data Bank (EMDB-29941) [https://www.ebi.ac.uk/emdb/EMD-29941], with corresponding coordinates in the Protein Data Bank (PDB: 8GCR) [https://www.rcsb.org/structure/unreleased/8GCR]. Complete gels and raw data for graphs are included in the 'Source Data File.xlsx'. MD simulation details are deposited in Google Cloud, accessible via the following link: [https://console.cloud.google.com/storage/e6ap_16e6_p53_complex_md_simulations]. This includes initial and final system configurations, trajectories, simulation parameters, and force fields for the five systems detailed in Supplementary Table 2.

Research involving human participants, their data, or biological material

Policy information about studies with human participants or human data. See also policy information about sex, gender (identity/presentation), and sexual orientation and race, ethnicity and racism.

Reporting on sex and gender	Not applicable
Reporting on race, ethnicity, or other socially relevant groupings	Not applicable
Population characteristics	Not applicable
Recruitment	Not applicable
Ethics oversight	Not applicable

Note that full information on the approval of the study protocol must also be provided in the manuscript.

Field-specific reporting

Please select the one below that is the best fit for your research. If you are not sure, read the appropriate sections before making your selection.

☒ Life sciences ☐ Behavioural & social sciences ☐ Ecological, evolutionary & environmental sciences

For a reference copy of the document with all sections, see nature.com/documents/nr-reporting-summary-flat.pdf

Life sciences study design

All studies must disclose on these points even when the disclosure is negative.

Sample size	Sample sizes are specified within the figure legends and Methods section. The sample size was not predetermined by statistical methods but was chosen to align with standard practices in the field. Quantitative experiments were typically replicated three times biologically. Additional replicates were conducted to reinforce the reliability of the measurements or when deemed practical for the system studied.
Data exclusions	In the ubiquitination assays for p53 and E6AP, one data point was omitted due to technical issues affecting sample loading and transfer efficacy. For experiments in quantifying the amount of HPV18E6 or E6AP in HeLa and HT1080 lysates, values were excluded if they fell outside the standard curve generated with recombinant MBP-18E6 or E6AP protein. Consistent with standard cryoEM procedures, particles that did not contribute to the classes of interest were excluded from the final reconstructions.
Replication	Replication details for each experiment are provided in the figure legends and Methods section. Experiments were typically conducted in triplicate, which is in line with standard practices in the field. All experiments were successfully replicated unless technical failures occurred, in which case controls also indicated failure.

Randomization

Randomization in this study was applied in cryoEM workflows, where particles were automatically divided into two random halves for separate refinement, a process implemented by cryoSPARC. For other experimental processes, randomization was not applicable as the study did not involve subjective decision-making regarding data inclusion, exclusion, or measurement.

Blinding

Investigators were not blinded to group allocation during the experiments or outcome assessment, since the analyses were based on quantitative endpoints that do not lend themselves to investigator bias.

Reporting for specific materials, systems and methods

We require information from authors about some types of materials, experimental systems and methods used in many studies. Here, indicate whether each material, system or method listed is relevant to your study. If you are not sure if a list item applies to your research, read the appropriate section before selecting a response.

Materials & experimental systems

n/a	Involved in the study
☐	☒ Antibodies
☐	☒ Eukaryotic cell lines
☒	☐ Palaeontology and archaeology
☒	☐ Animals and other organisms
☒	☐ Clinical data
☒	☐ Dual use research of concern
☒	☐ Plants

Methods

n/a	Involved in the study
☒	☐ ChIP-seq
☒	☐ Flow cytometry
☒	☐ MRI-based neuroimaging

Antibodies

Antibodies used

See Materials and Methods for additional details.

p53 Ubiquitination Assays:
α-p53: R&D Systems, Cat# MAB1355, Lot# IAH0422071, dilution 1:1,000.
Secondary (Goat anti-Mouse, 800 nm): LI-COR, Cat# 926-32210, Lot# D11116-25, dilution range 1:15,000 to 1:20,000.

EE6AP Ubiquitination Assays:
α-E6AP: Cell Signaling, Cat# D10D3, Lot# D21207-05, dilution 1:1,000.
Secondary (Goat anti-Rabbit, 680 nm): LI-COR, Cat# 926-6807, Lot# D21207-05.

Cellular Experiments:
HPV18 E6: Genetex, Cat# GTX132687, Lot# 42592, dilution 1:1,000.
HPV16 E6: Genetex, Cat# GTX132686, Lot# 44545, dilution 1:1,000.
Anti-E6AP: Sigma, Cat# E8655, Lot# 0000137246, dilution 1:1,000.
Anti-GAPDH: Thermo, Cat# MA5-15738, Lot# WL332983, dilution 1:1,000.

Secondary Antibodies for Cellular Experiments:
Donkey anti-Rabbit IRdye 800CW: LI-COR, Cat# 925-32213, Lot# D20119-01, dilution range 1:15,000 to 1:20,000.
Donkey anti-Mouse IRdye 680RD: LI-COR, Cat# 925-68072, Lot# D20125-11, dilution range 1:15,000 to 1:20,000.
Donkey anti-Mouse IRdye 800CW: LI-COR, Cat# 925-32212, Lot# D20412-01, dilution range 1:15,000 to 1:20,000.

Validation

Commercially sourced antibodies were validated by their respective suppliers. Additional validations conducted in this study are described as follows:

α-p53 (R&D Systems, Cat# MAB1355, Lot# IAH0422071): Diluted 1:1,000. Validated for Western blot, immunoprecipitation, and immunocytochemistry. Detects human, mouse, and rat p53.

α-E6AP (Cell Signaling, Cat# D10D3, Lot# D21207-05): Diluted 1:1,000. Validated for Western blot. Recognizes human, mouse, rabbit, and monkey E6AP.

α-HPV18 E6 (Genetex, Cat# GTX132687, Lot# 42592): Diluted 1:1,000. Validated for Western blot. Specificity confirmed in various cell lines and by siRNA knockdown (Suppl. Fig. 27).

α-HPV16 E6 (Genetex, Cat# GTX132686, Lot# 44545): Diluted 1:1,000. Validated for Western blot. Specificity tested in multiple cell lines (Suppl. Fig. 27).

α-E6AP (Sigma, Cat# E8655, Lot# 0000137246): Diluted 1:1,000. Validated for various applications. Recognizes E6AP in several species. Confirmed by siRNA knockdown (Suppl. Fig. 27).

α-GAPDH (Thermo, Cat# MA5-15738, Lot# WL332983): Diluted 1:1,000. Validated for numerous applications. Recognizes GAPDH across a range of species.

Eukaryotic cell lines

Policy information about cell lines and Sex and Gender in Research

Cell line source(s)	All cell lines were obtained form ATCC. Catalog and lot numbers are as follows:<br>HT1080: ATCC, cat. no. CCL-121, lot # 70032964<br>HeLa: ATCC, cat. no. CCL-2, lot # 70033477<br>CaSki: ATCC, cat. no. CRM-CRL-1550, lot # 70032964
Authentication	None of the cell lines were internally authenticated after buying form ATCC where STR profiling was conducted.
Mycoplasma contamination	Cell lines in use are tested for mycoplasma contamination on a monthly basis.
Commonly misidentified lines<br>(See ICLAC register)	CaSki, HeLa and HT1080 cells were not reported as a commonly misidentified cell lines (ICLAC, version 12). However, HeLa cells have been reported as a contaminating cell line in multiple other cell lines (ICLAC, version 12).
